# Multi-omics approach reveals the contribution of KLU to leaf longevity and drought tolerance

**DOI:** 10.1093/plphys/kiaa034

**Published:** 2020-11-28

**Authors:** Liang Jiang, Takuya Yoshida, Sofia Stiegert, Yue Jing, Saleh Alseekh, Michael Lenhard, Francisco Pérez-Alfocea, Alisdair R Fernie

**Affiliations:** 1 Max-Planck-Institut für Molekulare Pflanzenphysiologie, Wissenschaftspark Golm, Am Mühlenberg 1, 14476 Potsdam, Germany; 2 Department of Genetics, University of Potsdam, 14469 Potsdam, Germany; 3 Department of Plant Nutrition, CEBAS-CSIC, Campus Universitario de Espinardo, 30100 Murcia, Spain

## Abstract

KLU, encoded by a cytochrome P450 CYP78A family gene, generates an important—albeit unknown—mobile signal that is distinct from the classical phytohormones. Multiple lines of evidence suggest that KLU/KLU-dependent signaling functions in several vital developmental programs, including leaf initiation, leaf/floral organ growth, and megasporocyte cell fate. However, the interactions between KLU/KLU-dependent signaling and the other classical phytohormones, as well as how KLU influences plant physiological responses, remain poorly understood. Here, we applied in-depth, multi-omics analysis to monitor transcriptome and metabolome dynamics in *klu*-mutant and *KLU*-overexpressing Arabidopsis plants. By integrating transcriptome sequencing data and primary metabolite profiling alongside phytohormone measurements, our results showed that cytokinin signaling, with its well-established function in delaying leaf senescence, was activated in *KLU*-overexpressing plants. Consistently, *KLU*-overexpressing plants exhibited significantly delayed leaf senescence and increased leaf longevity, whereas the *klu*-mutant plants showed early leaf senescence. In addition, proline biosynthesis and catabolism were enhanced following *KLU* overexpression owing to increased expression of genes associated with proline metabolism. Furthermore, *KLU*-overexpressing plants showed enhanced drought-stress tolerance and reduced water loss. Collectively, our work illustrates a role for KLU in positively regulating leaf longevity and drought tolerance by synergistically activating cytokinin signaling and promoting proline metabolism. These data promote KLU as a potential ideal genetic target to improve plant fitness.

## Teaser

A cytochrome P450, KLU, contributes to leaf longevity and drought tolerance by regulating cytokinin signaling and proline metabolism.

## Introduction

As sessile organisms, plants have several adaptive mechanisms allowing them to cope with fluctuating environmental conditions. Among these, leaf senescence greatly increases plant fitness, reproduction, and survival by actively recycling nutrients and energy from old leaves into newly developing organs or offspring (Yoshida, 2003; Lim et al., 2007). Naturally, leaf senescence occurs in an age-dependent manner, and its related processes, including chlorophyll degradation and programmed cell death, are finely tuned by the integration of cues from both exogenous stimuli, such as nutrient availability and biotic/abiotic stress, and endogenous developmental signals, including age, reactive oxygen species (ROS), reproduction transition, and phytohormones (Nam, 1997; Lim et al., 2003; Guo and Gan, 2005; Woo et al., 2013). Among the phytohormones, abscisic acid (ABA; Zhao et al., 2016; Mao et al., 2017), ethylene (Koyama, 2014), salicylic acid (SA; Zhang et al., 2013, 2017), jasmonate (JA; Hu et al., 2017), and strigolactones (SLs; Woo et al., 2001; Ueda and Kusaba, 2015) have positive effects on the promotion of leaf senescence, whereas cytokinin has a well-defined function in delaying leaf senescence (Zwack and Rashotte, 2013).

The biosynthesis of cytokinins begins with *ISOPENTENYL TRANSFERASE* (*IPT*) genes (Kakimoto, 2001; Takei et al., 2001; Sakakibara, 2006), encoding rate-limiting enzymes that catalyze the reaction of adding a prenyl group to ADP/ATP to produce *N*^6^-(Δ2-isopentenyl) adenine (iP) ribotides. iP ribotides can subsequently be converted to *trans*-zeatin (*t*Z) type ribotides by the cytochrome P450 enzymes CYP735A1 and CYP735A2 (Takei et al., 2004; Kiba et al., 2013). In the last step, the LONELY GUY (LOG) protein catalyzes the reaction that converts cytokinin from an inactive form to an active form (Kurakawa et al., 2007; Kuroha et al., 2009). In addition to *de novo* biosynthesis affecting the final content of cytokinins, the levels of active cytokinins can be modulated via conjugation to a sugar (Brzobohatý et al., 1993), as well as through irreversible cleavage by CYTOKININ OXIDASEs (CKXs; Werner et al., 2003). The cytokinin signal transduction pathway is mediated by a two-component-like system in which the cytokinin receptors *Arabidopsis* HISTIDINE KINASEs (AHKs) pass the phosphoryl groups to AUTHENTIC HISTIDINE PHOSPHOTRANSFERASEs (AHPs) which passes it to A RESPONSE REGULATOR (RR or ARR; Hutchison and Kieber, 2002; Hwang et al., 2012; Kieber and Schaller, 2014). In *Arabidopsis*, there are two types of ARRs involved in cytokinin signaling: type-A and type-B ARRs. Type-B ARR transcription factors are essential for the initial transcriptional response to cytokinins (Brandstatter and Kieber, 1998; D’Agostino et al., 2000). By contrast, type-A ARRs lack a transcriptional regulatory domain and act as negative-feedback regulators of cytokinin signaling (Hwang and Sheen, 2001; El-Showk et al., 2013). In addition to ARRs, CYTOKININ RESPONSE FACTOR (CRF), belonging to the APETALA2 (AP2) transcription factor family, has been identified as a novel class of response regulators of cytokinin (Rashotte et al., 2006).

Delayed leaf senescence mediated by cytokinins has been studied for many decades (Zwack and Rashotte, 2013). Pioneering research from Rihmond and Lang (1957) showed that cytokinin analog kinetin treatment delayed the loss of chlorophyll and extended leaf life-span in *Xanthium pennsylvanicum*. Since then, the cytokinin effects on delaying leaf senescence have been found in many other species (Dyer and Osborne, 1971; Gan and Amasino, 1996). Another key evidence supporting the idea that cytokinins delay leaf senescence is a strong negative correlation between cytokinin amount and leaf senescence progress (Khan et al., 2014). Transcriptome analysis in *Arabidopsis* showed that expression levels of cytokinin biosynthesis genes were dramatically decreased in senescent leaves (Buchanan-Wollaston et al., 2005). This phenomenon led to the design of a system in which the *IPT* gene is expressed under the promoter of *SENESCENCE ASSOCIATED GENE 12* (*SAG12*), a reference gene for characterizing leaf senescence (Gan and Amasino, 1995). This *PROSAG12:IPT* system has been applied to many important crop species including rice (*Oryza sativa*), tomato (*Solanum lycopersicum*), alfalfa (*Medicago sativa*), cauliflower (*Brassica oleracea var. botrytis*), wheat (*Triticum aestivum*), and cotton (*Gossypium hirsutum*), all of which showed delay leaf senescence (McCabe et al., 2001; Lin et al., 2002; Calderini et al., 2007; Nguyen et al., 2008; Sykorová et al., 2008; Ma and Liu, 2009; Liu et al., 2012). Besides the effect of cytokinin contents on leaf senescence, the component of cytokinin signaling-mediated senescence regulation was revealed by the identification of the gain-of-function mutation in a histidine kinase cytokinin receptor AHK3, which showed delay leaf senescence (Kim et al., 2006). Constitutive activation of *AHK3* leads to phosphorylation on the Asp-80 residue of a B-type response regulator ARR2. Similar to the gain-of-function *ahk3* phenotype, overexpression of *ARR2* also resulted in delayed leaf senescence during dark-induced and age-dependent senescence (Kim et al., 2006). Another type of CRF, CRF6, has been found to negatively regulate developmental senescence and may have a similar role in response to stress (Zwack et al., 2013). It has been proposed that AHK3-mediated cytokinin signaling activates ARR2 and induces *CRF6*, resulting in activation of extracellular invertase (Zwack and Rashotte, 2013), which is essential for the cytokinin-mediated delay of senescence as it supplies carbohydrates to sink tissues (Balibrea Lara et al., 2004).

In contrast to the decreased contents of cytokinin during leaf senescence, the amount of ABA, SA, and JA is increased as senescence progresses (Khan et al., 2014). Both ABA and SA have long been known to play positive roles in leaf senescence (Becker and Apel, 1993; Morris et al., 2000). ABA can induce plant senescence via regulating the expression of some *SAGs* (Weaver et al., 1998). The relationship between SA and leaf senescence was revealed by analysis of mutants disrupted in SA catabolism and signaling components, such as SA3-HYDROXYLASE (S3H; Zhang et al., 2013), PROTEIN S-AXYLTRANSFERASE14 (PAT14; Zhao et al., 2016), NONEXPREESSION OF PR GENES1 (NPR1; Yoshimoto et al., 2009), and PHYTOALEXIN DEFICIENT4 (PAD4; Vogelmann et al., 2012). All these mutants accumulating high levels of SA display precocious leaf senescence. Consistent with this, transcriptome analysis revealed a high degree of overlap in the transcription patterns between SA treatment and leaf senescence (Buchanan-Wollaston et al., 2005).

Besides phytohormones, nutrition limitation, especially in the case of nitrogen, has been shown to aggravate leaf senescence (Diaz et al., 2008; Agüera et al., 2010). As such, leaf senescence is understood as indispensable metabolic adjustments for nutrient recycling and remobilization, which affects both carbon and nitrogen management (Bleecker, 1998). A large number of genes are differentially expressed during leaf senescence, including genes in nitrate transport, nitrogen assimilation and remobilization, proteolysis, and autophagy (Havé et al., 2017), indicating that increased protein degradation facilitates the recycling of protein reserves in leaves.

The *Arabidopsis* cytochrome P450 gene *KLU* (also known as *CYP78A5*) is predicted to produce a mobile molecule that promotes the growth of floral organs and leaves in a non-cell-autonomous manner (Anastasiou et al., 2007; Adamski et al., 2009; Kazama et al., 2010). Given that *klu* mutants showed a higher rate of leaf initiation, as well as smaller leaf, sepal, and petal sizes due to their reduced cell number (Anastasiou et al., 2007; Wang et al., 2008), KLU was suggested to be a positive regulator of cell proliferation, which is a process governed by cytokinin. The early transcriptional response to *KLU* activity is distinct from that of classical phytohormones, supporting the idea that KLU belongs to a separate signaling pathway regulating organ size (Anastasiou et al., 2007). In *Arabidopsis*, *KLU* is expressed in the inner integument of developing ovules and stimulates cell proliferation, thereby contributing to seed size (Adamski et al., 2009). Similarly, the ectopic overexpression of *KLU* homologs in a wide variety of plant species, particularly in crops, also leads to large seed or fruit sizes (Fang et al., 2012; Chakrabarti et al., 2013; Yang et al., 2013; Ma et al., 2015), partly because of reduced fertility (Fang et al., 2012). Owing to its agricultural importance, functional characterization of *KLU* has attracted much attention. In this study, we demonstrated that *Arabidopsis* KLU plays positive roles in delaying leaf senescence. The combination of transcriptome analysis and metabolite profiling revealed that KLU contributes to leaf longevity by synergistic activation of cytokinins signaling. A signature metabolic change among wild-type (WT), *klu*-mutant, and *KLU*-overexpressing plants is the accelerated metabolic flux from glutamate to proline. As proline is a well-defined protective factor in plants coping with many stress conditions, the overexpression of *KLU* was associated with enhanced resistant to drought tolerance and dark-triggered leaf senescence. The expression of proline biosynthesis genes increased following both *KLU* overexpression and cytokinin treatment. Taken together, our research reveals the molecular mechanism through which KLU contributes to leaf senescence and drought tolerance.

## Results

### Transcriptome analysis and hormone measurement reveal that KLU/KLU-dependent signaling has complex influences on phytohormones

**Figure 1:**
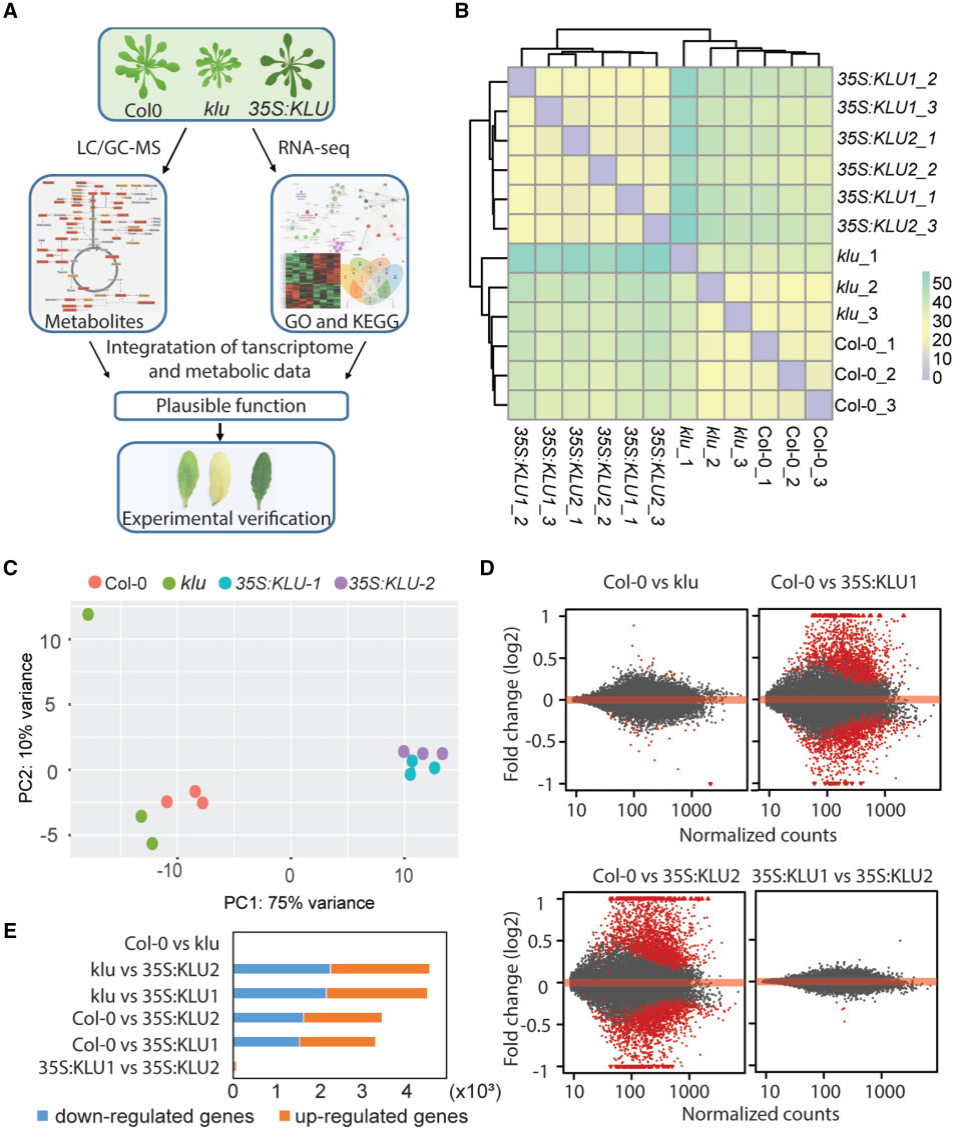
Overview of the transcriptome changes. (**A**) Schematic representation of experimental strategy. The images of rosettes and leaves were digitally extracted for comparison among the genotypes. (**B**) A sample hierarchical clustering based on the heatmap of sample–sample distances. Distance heatmap computed from the count matrix. (**C**) Principal component plot of the individual samples based on count matrix. These percentages do not add up to 100% because there are more dimensions that contain the remaining variance. (**D**) MA-plot showing log2 fold change (*y*-axis) of a particular comparison over the mean of normalized counts (*x*-axis) for all the samples. Red points indicate adjusted *P*-value is <0.05. Points falling out of the window are plotted as triangles pointing either up or down. (**E**) Number of DEGs in different group samples. Yellow boxes, up-regulated genes, blue boxes, down-regulated genes.

**Figure 2:**
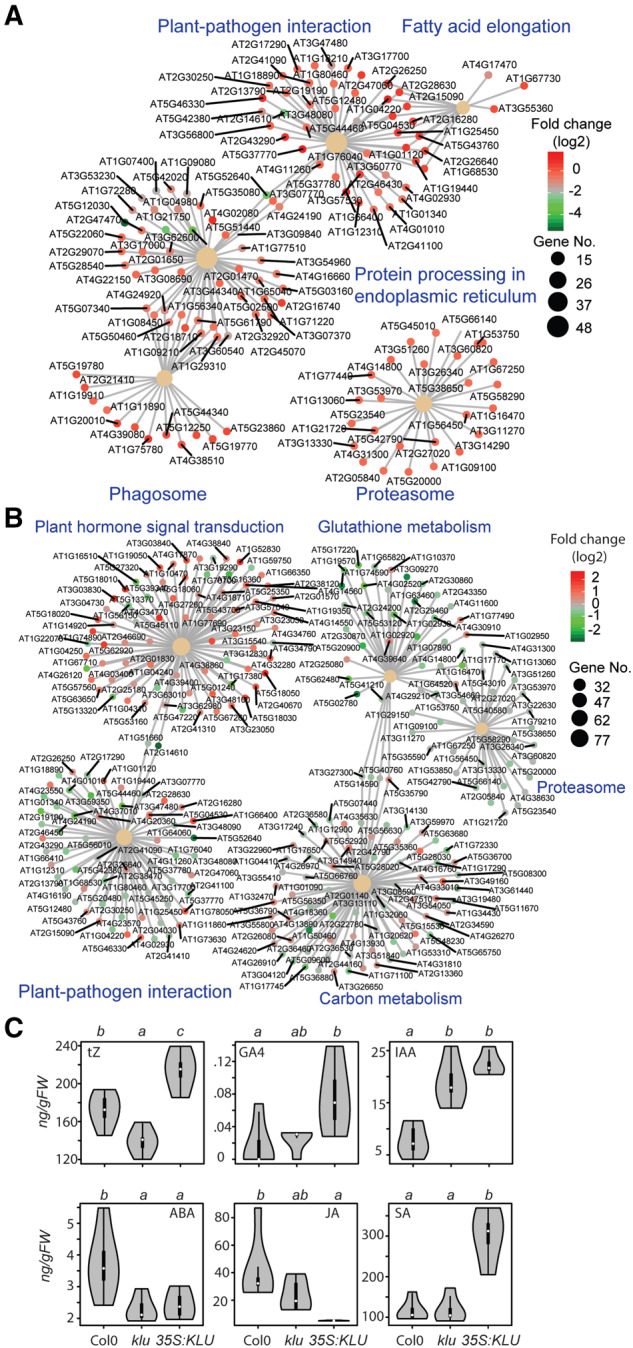
KEGG enrichment analysis of DEGs and phytohormone profiling. (**A**) KEGG enrichment analysis of DEGs of WT versus OE. (**B**) KEGG enrichment analysis of DEGs of *klu* versus OE. The circle size of each group scaled from the gene number enriched in each group, the dot colors indicate log2 fold change of WT/*klu* compared with OE. (**C**) *trans*-Zeatin (*t*Z), GA4, IAA, ABA, JA, and SA contents in the leaves of WT, *klu*, and *35S:KLU* plants at 35 DAG. *n* = 4–6, ANOVA, Tukey’s HSD, *P* < 0.05.

To systematically elucidate how KLU/KLU-dependent signaling influences cell physiology through interacting with other phytohormone signaling pathways, a comprehensive multi-omics analysis was applied to reveal alterations in the transcriptome, phytohormones, and primary metabolites across WT (Col-0), *klu*-mutant, and *KLU*-overexpressing plants (Figure 1, A). To get an overview of the transcriptomic data, sample clustering based on the read count matrix was conducted, resulting in two major clusters, one between *KLU*-overexpression lines and another between WT and the *klu* mutant (Figure 1, B). Principal component analysis (PCA) based on the count matrix of all genes across three genotypes showed clear differentiation of the two independent overexpression lines with WT and the *klu* mutant, in which the first principal component (PC1) and PC2 explain 75% and 10% of the variance, respectively (Figure 1, C). After filtering out the low-read counts, differentially expressed genes (DEGs) were analyzed by DESeq2 with adjusted *P*-value < 0.05. MA plots showed that a high number of DEGs were detected when comparing *KLU*-overexpression lines with both WT and the *klu* mutants, whereas very few DEGs were found between WT and *klu* (Figure 1, D, Supplemental Figure S1, A, and Supplemental Table S1). A total of 3,284 DEGs and 3,434 DEGs were found between WT and two different *35S:KLU* lines, respectively (Figure 1, E). By contrast, 4,476 DEGs and 4,525 DEGs were found between the *klu* mutant and the two *KLU*-overexpression lines, respectively (Figure 1, E). Only 59 DEGs were identified between WT and *klu* (Figure 1, E and Supplemental Figure S1, B). The few number of DEGs between WT and *klu* might be caused by functional redundancy in the *KLU* gene family (Bak et al., 2011; Fang et al., 2012). Reverse transcription quantitative PCR (RT-qPCR) revealed that the expression levels of several other members of this family were increased in the *klu* mutant, whereas they were decreased in *35S:KLU1* plants (Supplemental Figure S1, C). Only three DEGs were found from a total of ∼21,000 expressed genes between the two independent *KLU*-overexpression lines. Therefore, we chose the common DEGs that were shared by the two independent *KLU*-overexpression lines compared with WT/*klu* (named Col-0 versus OE and *klu* versus OE, respectively) for later gene ontology (GO) analysis and kyoto encyclopedia of genes and genomes (KEGG) pathway enrichment analysis. Both Col-0 versus OE DEGs and *klu* versus OE DEGs showed similar GO terms enrichment related to systemic acquired resistance, cell death, and nitrogen compound metabolic process (Supplemental Figure S2). In KEGG pathway enrichment analysis, both DEGs shared common pathways pertaining to plant–pathogen interaction and proteasome, which also can be traced from GO term analysis (Figure 2, A and B). Following comparison of KEGG pathway enrichment between Col-0 versus OE DEGs and *klu* versus OE DEGs, we observed that phytohormone signaling transduction and carbon metabolism were enriched in *klu* versus OE DEGs but not in the Col-0 versus OE DEGs (Figure 2, A and B). Among the key genes in phytohormone metabolism, transport, perception, and signaling (Supplemental Tables S2–S9), many showed altered expression, especially in the comparison *klu* versus OE (Supplemental Figures S3 and S4). In addition, phytohormone profiling showed that the major detectable phytohormones were altered in the *KLU*-overexpressing line. The *t*Z of cytokinin, GA4, IAA, and SA accumulated in the *KLU* overexpressor, whereas ABA and JA decreased (Figure 2, C).

### Cytokinin signaling is altered following *KLU* overexpression

**Figure 3:**
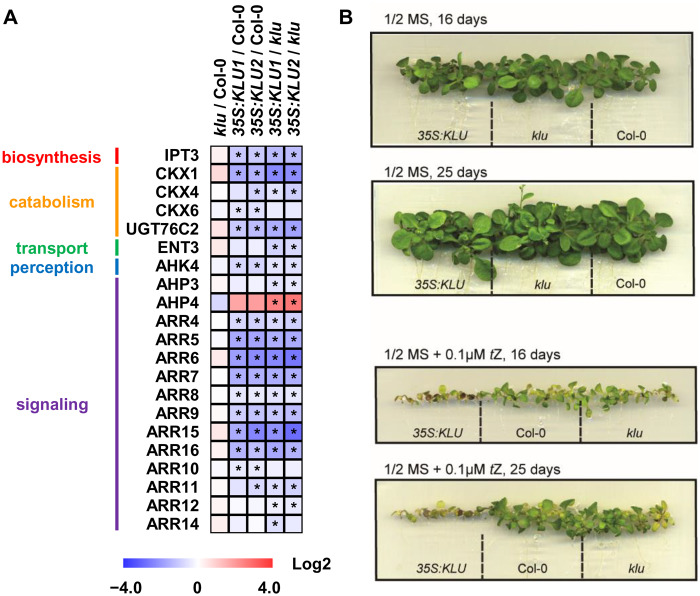
Cytokinin signaling pathway altered in *KLU*-overexpressors. (**A**) The expression of the genes associated with cytokinin metabolism, transport, perception, and signaling in *klu* and *35S:KLU* plants analyzed by RNA-sequencing. Asterisks indicate the DEGs (adjusted *P*-value < 0.05). The detailed expression data are available in Supplemental Table S2. (**B**) Phenotypical response of WT, *klu*, and *35S:KLU* upon 0.1-µM *t*Z treatment at the stage suggested in the figure. *n* = 15.

Among the phytohormone changes in the *klu* mutant and *KLU* overexpressors, cytokinin particularly drew our attention because of the following facts. First, the contents of *t*Z of cytokinin were decreased and increased in *klu*-mutant and *KLU*-overexpressor plants, respectively, whereas other hormones did not show opposite changes between the mutant and the overexpressor (Figure 2, C). Second, our transcriptome data and RT-qPCR analysis revealed that several cytokinin-related genes, including *CKX1*, *CKX4*, and type-A *ARRs*, were down-regulated in the *KLU* overexpressors, whereas *AHP4* was up-regulated (Figure 3, A and Supplemental Figure S4, A). Among six functional cytokinin oxidase genes in *Arabidopsis* (Kieber and Schaller, 2014), *CKX1* and *CKX4* showed significant changes in the comparison *klu* versus OE. Thus, the down-regulation of cytokinin catabolism genes *CKX1* and *CKX*4 in the *35S:KLU* plants could be one of the reasons for the high level of cytokinins. Third, consistent with phytohormone profiling results, the *35S:KLU* plants also showed hypersensitivity to the *t*Z-type cytokinin treatment compared with the WT and the *klu* mutants (Figure 3, B).

### KLU/KLU-dependent signaling influences leaf aging

**Figure 4:**
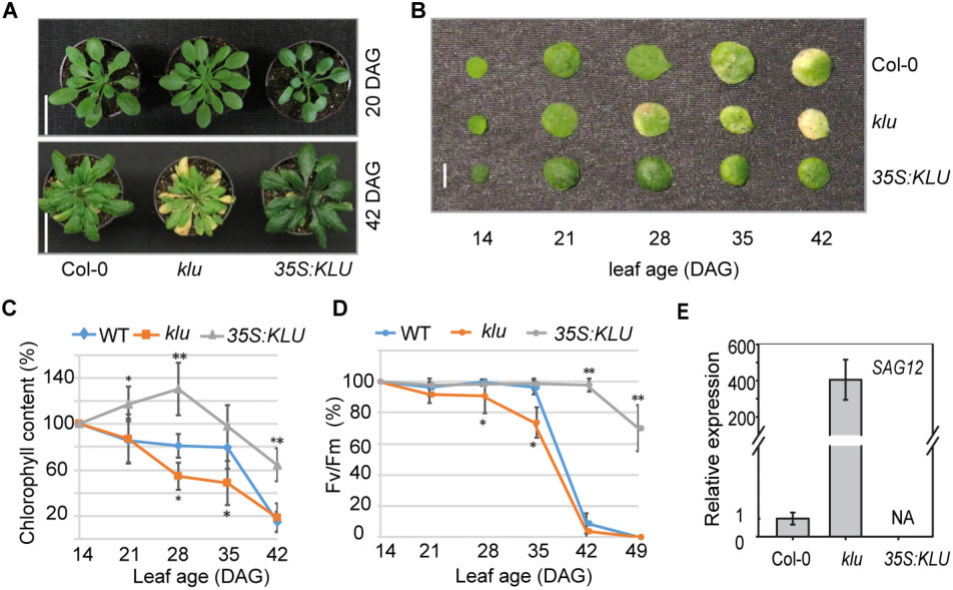
The *Arabidopsis KLU* contributes to leaf longevity. (**A**) Representative phenotypes of WT, *klu*, and *35S:KLU* plants at different stages. The stems were removed at the 42-DAG stage. Scale bar, 3 cm. (**B**) Representative phenotypes of the third leaf of WT, *klu*, and *35S:KLU* plants at different stages. Scale bar, 0.5 cm. (**C**) The chlorophyll contents in the third leaf of WT, *klu*, and *35S:KLU* leaves at different ages. Error bars represent sd (*n* = 14). (**D**) Photochemical efficiency (Fv/Fm) in the third leaf of WT, *klu*, and *35S:KLU* leaves were examined at different ages. Error bars represent sd (*n* = 16–20). (**E**) Expression of the molecular senescence marker gene *SAG12* in the third leaf of WT, *klu*, and *35S:KLU* plants at 28 DAG. Error bars represent sd (*n* = 3). The *Arabidopsis UBIQUITIN 5* was used as internal control. Plant materials were grown under a 16-h light/8-h dark cycle. Asterisks indicate statistically significant differences when compared with WT by Student’s *t* test (*P < 0.05, **P < 0.01).

We reasoned that either the *klu* mutant or *KLU* overexpressors can share some common phenotypic features that have been revealed in cytokinin signaling-defective mutants. One of these phenotypic features we observed was leaf senescence; it is well known that an increase in cytokinin contents or the activation of cytokinin signaling greatly prolongs leaf longevity (Zwack and Rashotte, 2013). The *klu* mutant displayed early yellowish leaves in the reproductive stage compared with WT (Figure 4, A), whereas the leaves of the *35S:KLU* plants showed darker green coloring compared with WT through the whole plant life cycle (Figure 4, A and Supplemental Figure S5, A). In *Arabidopsis*, many early- or delayed-senescence mutants were reported to show early- or delayed-flowering, respectively (Wingler, 2011). Similarly, the early-senescence *klu* mutant also showed early floral transition, small floral organs, and accelerating leaf initiation (Supplemental Figure S5, B–F). To examine leaf senescence phenotypes in detail, phenotypical examination of the third leaf throughout their life spans showed that the *klu* mutant had an early senescence phenotype, whereas *35S*:*KLU* plants had greatly delayed leaf senescence (Figure 4, B). The photosynthetic pigment concentration and photochemical efficiency of photosystem II (Fv/Fm), two critical physiological markers related to leaf senescence, were determined in the third leaf at different growth stages. As shown in Figure 4, C, higher levels of chlorophyll were maintained in *35S:KLU* compared with WT and *klu* plants, whereas a dramatically decreased chlorophyll content was observed in the *klu* mutant at the later stage. Similarly, there was a significant reduction of Fv/Fm in the *klu* mutant compared with WT and *35S:KLU* (Figure 4, D). In addition, the expression of *SAG12*, a marker gene for leaf senescence, was increased in the *klu* mutant at 28 d after germination (DAG), whereas no detectable transcripts were found in *35S:KLU* plants at the same stage (Figure 4, E). These results indicate that *KLU* plays positive roles in leaf longevity.

### Nitrate transport and nitrogen and proline metabolism are altered in *KLU* overexpressors

**Figure 5:**
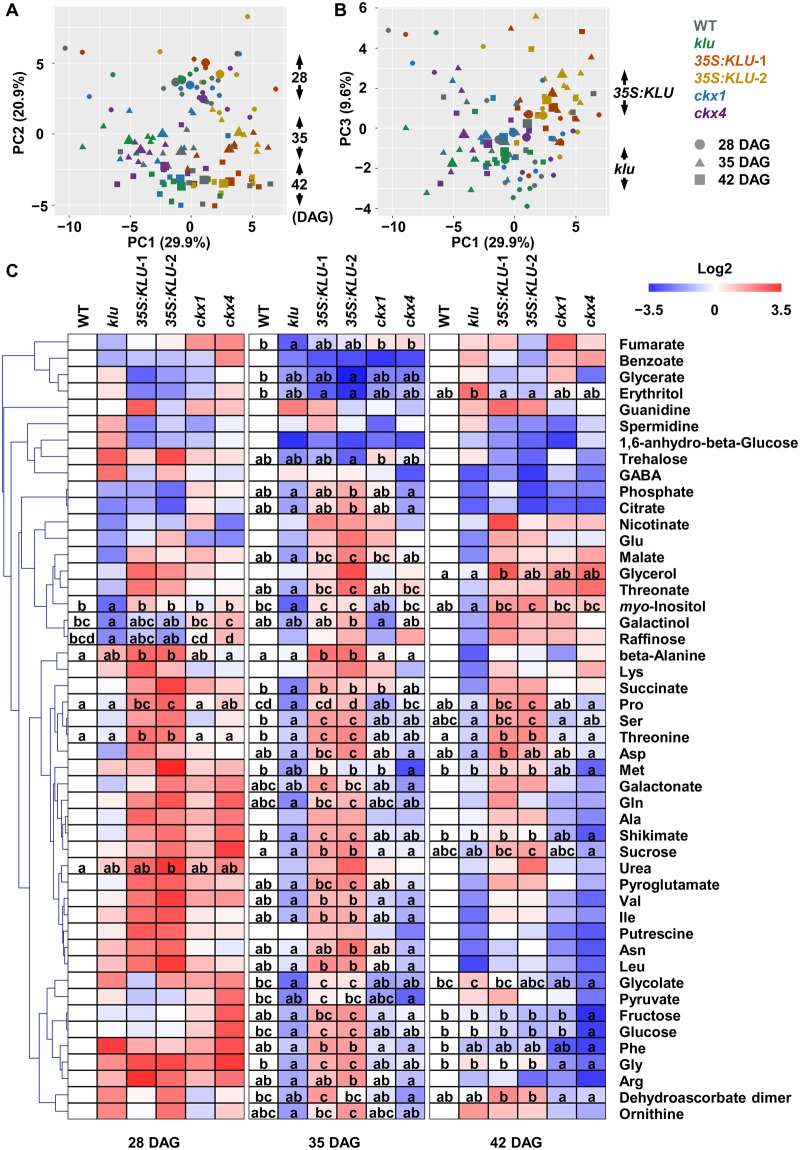
The metabolite profile of *KLU*-overexpressing lines during vegetative growth. Primary metabolites extracted from aerial parts of plants grown on soil for 28, 35, and 42 DAG were analyzed by GC–MS. Chromatograms and mass spectra were evaluated using Chroma TOF 1.0 (LECO) and Tag Finder 4.0 (Luedemann et al., 2012), and 48 metabolites were annotated. Data comprise five or six biological replicates. (**A**) and (**B**) PCA was performed by using R software. The mean points of replicates are indicated by larger symbols. (**C**) Heat map showing relative accumulation of each metabolite compared with those in WT plants. For each metabolite, the value of the corresponding WT (28, 35, and 42 DAG) was set to 0. Different letters denote statistically significant differences (*P* < 0.05) within each time point by one-way ANOVA followed by a Tukey’s *post hoc* test.

Besides phytohormone signaling pathways, our KEGG pathway enrichment analysis indicated that the carbon metabolism-related genes were differentially expressed in the *KLU* overexpressors (Figure 2). To reveal the role of *KLU* in redistributing nutrient and metabolic flow, we profiled the primary metabolites by gas chromatography–mass spectrometry (GC–MS) to identify changes in metabolite levels among WT, *klu*, and *35S*:*KLU* plants during vegetative growth (Figure 5). To assess secondary effects potentially caused by the altered leaf senescence (Figure 4), the whole rosettes were harvested at 35 DAG, the same conditions for the transcriptome analysis (Figure 1), and two additional time points (28 and 42 DAG). Furthermore, to examine the effects of decreased expression of *CKX*s (Figure 3) on metabolite profiles, the *ckx1-1* and *ckx4-1* mutants (Supplemental Figure S6), hereafter *ckx1* and *ckx4*, were also analyzed. In total, 48 primary metabolites were annotated, and subsequent PCA revealed that the samples were grouped by the harvesting dates (Figure 5, A). Moreover, *klu* and *35S:KLU* plants were separated by PC1 and PC3 (Figure 5, B), implying that the differences between *klu* and *35S:KLU* plants were clearer than those between WT and *35S:KLU* plants. Although several metabolites, including galactinol and raffinose, contributed to the separation of the samples during growth, as indicated in PC2 (Figure 5, A and Supplemental Figure S7, A), these metabolites did not show significant changes between *klu* and *35S:KLU* plants (Figure 5, C). Therefore, we focused on the metabolic changes within the same time point. Many metabolites showed significant changes among the lines at 35 DAG (Figure 5, C and Supplemental Figures S8–S12), whereas several metabolites, such as threonine, proline, and *myo*-inositol, showed constitutive accumulation in *35S:KLU* plants compared with the *klu* mutant (Supplemental Figure S8). Given than the accumulation patterns of these metabolites during growth were comparable among the lines (Supplemental Figure S8, B), the overexpression of *KLU* might directly affect the biosynthetic and catabolic pathways of these metabolites. Interestingly, sucrose, glucose, and fructose showed transient accumulation at 35 DAG in *35S:KLU* plants compared with the *klu* mutant, whereas glucose and fructose contents in the *klu* mutant increased to similar levels as in *35S:KLU* plants at 42 DAG (Supplemental Figure S12). These results imply that sugar accumulation during vegetative stages was enhanced by the overexpression of *KLU*. The profiles of the *ckx* mutants were comparable with that of WT; however, several metabolites, including amino acids and sugars, accumulated less in the *ckx4* mutant, especially at 42 DAG, albeit to a lesser extent in the *ckx1* mutant (Figure 5, C).

The metabolomic and transcriptomic data were further integrated to explore metabolic changes, which were associated with transcriptional changes. The GO category nitrogen compound metabolic process was one of the enriched categories (Supplemental Figure S2), and indeed the genes associated with nitrate transport, ammonium transport, and nitrogen assimilation were differentially expressed in *35S:KLU* plants (Supplemental Figure S13, A–C and Supplemental Table S10). The altered expression of these genes might be related to increased levels of arginine and ornithine (Figure 5, C), which accumulated under nitrogen-limited conditions (Tschoep et al., 2009). In addition to arginine and ornithine, their derivative, proline, showed constitutive accumulation in *35S:KLU* plants (Figure 5, C), and thus the expression of the genes associated with proline metabolism was displayed with the metabolic pathways (Supplemental Figure S13, D and Supplemental Table S11). The increased expression of the genes for arginase (*ARGAH2*) and *N*-acetylornithine deacetylase (*NAOD*) and those in proline biosynthesis and catabolism, except for *ProDH1*, might be related to the accumulation of ornithine and proline, respectively. Several genes in glutamate metabolism also showed altered expression in *35S:KLU* plants; however, we did not observe any significant changes in the level of 4-aminobutyric acid (GABA; Figure 5, C) and could not annotate 2-oxoglutarate in our chromatograms. Collectively, the genes in nitrate and ammonium transport, nitrogen assimilation, and proline metabolism were differentially expressed by the overexpression of *KLU*, and this altered gene expression might be associated with increased levels of arginine, ornithine, and proline.

### Overexpression of *KLU* enhances drought tolerance

**Figure 6:**
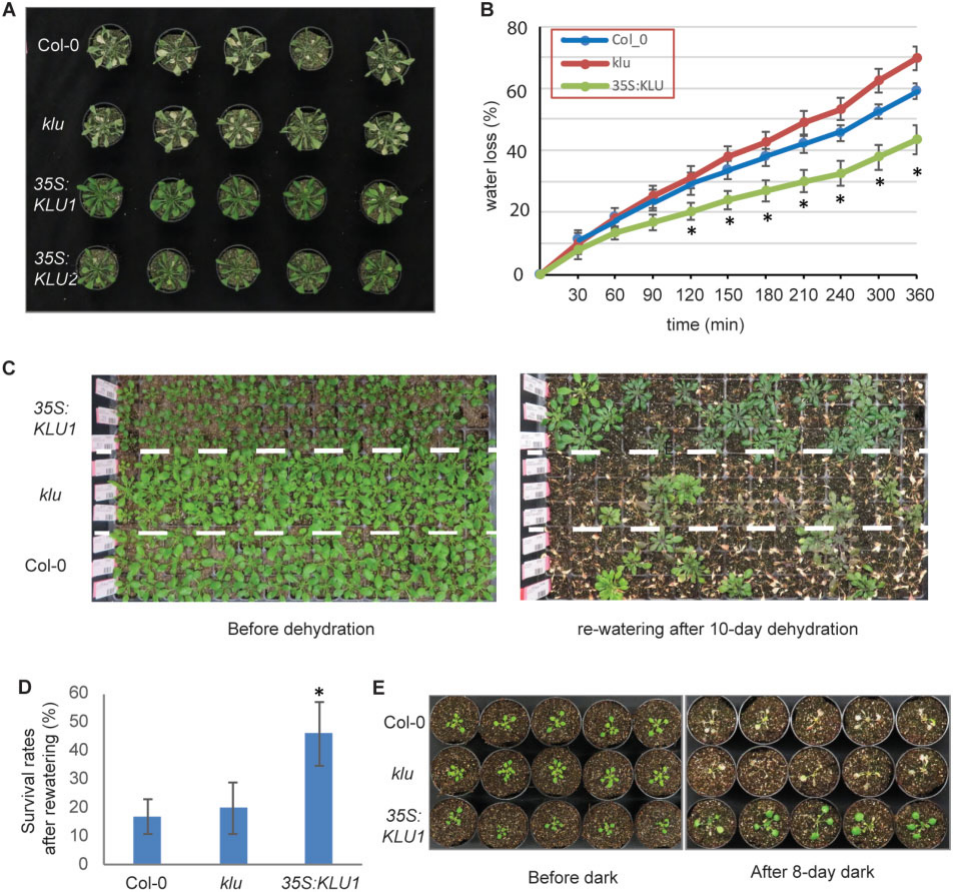
Overexpression of *KLU* contributes to drought tolerance. (**A**) Overexpression of *KLU* corresponds with strong drought-stress tolerance. Twenty-one-day-old seedlings were subjected to drought conditions for 7 d. *n* = 14. (**B**) Time-course water loss of the detached whole aerial part of 21-d-old WT, *klu*, and *35S:KLU* seedlings. Error bars represent sd (*n* = 12). Student’s *t* test (*P < 0.05). (**C**) 14-d-old seedlings (left) were subjected to drought conditions by withholding water for 10 d and then re-watered. Photographs were taken 4 d after re-watering (right). *n* = 120. (**D**) The survival rate after re-watering in C. Error bars represent sd. Student’s *t* test (*p < 0.05). (**E**) 14-d-old seedlings (left) were subjected to dark treatment for 7 d and recovered for 2 d under 8-h/16-h dark/light conditions (right). *n* = 10.

Previous studies have shown that cytokinin signaling plays negative roles in drought tolerance (Nishiyama et al., 2011, 2013; Salleh et al., 2016). However, delayed senescence via increased cytokinin contents could enhance drought tolerance (Rivero et al., 2007). Our phytohormone measurement and transcriptomic analysis showed increased levels of cytokinins and activation of cytokinin signaling in *KLU*-overexpressing plants. We further examined whether the overexpression of *KLU* affected drought tolerance. After drought stress for 7 d, *KLU*-overexpressing plants showed increased drought-stress tolerance compared with WT (Figure 6, A). *KLU*-overexpressing plants showed greenish leaves, whereas WT and *klu* plants exhibited yellowish leaves. Furthermore, the rate of water loss was lower in *KLU*-overexpressing plants compared with WT after 150-min dehydration (Figure 6, B). Consistently, stomatal apertures of *35S:KLU* plants were slightly closed even after incubation in the stomatal opening buffer, although ABA-responsive stomatal closure was not observed in the overexpressing plants (Supplemental Figure S14). After growing under drought-stress conditions for 10 d, we re-watered the plants for 4 d and monitored their survival rates. The *35S:KLU* plants showed decreased sensitivity to drought stress compared with WT, whereas the *klu* mutant displayed comparable characteristics with WT under the stress conditions (Figure 6, C and D). In addition, darkness is one of the most potent external stimuli that accelerates leaf senescence (Liebsch and Keech, 2016). *35S*:*KLU* plants exhibited greatly delayed dark-induced leaf senescence (Figure 6, E), suggesting that the overexpression of *KLU* leads to significant tolerance of carbon starvation. Collectively, these results indicate that *KLU* overexpression both affects leaf senescence and has positive effects on drought-stress tolerance.

## Discussion

In land plants, leaf senescence occurs naturally in an age-dependent manner and is influenced by the integration of internal and environmental signals, and numerous components have been identified in the associated complex regulatory mechanisms (Lim et al., 2007; Guo, 2013; Kim et al., 2016; Yolcu et al., 2018). In this study, our results unveiled that KLU acts as a negative regulator of leaf senescence, delaying the progress of leaf senescence via alteration of phytohormone metabolism and signaling (Figure 4 and Supplemental Figure S5). Transcriptomic analysis suggested that KLU activates the cytokinin signaling pathway by synergistically controlling cytokinin homeostasis and cytokinin signaling (Figure 3). Furthermore, metabolite profiling together with transcriptome data revealed that nitrogen assimilation and proline metabolism were enhanced in *KLU*-overexpression lines (Figure 5). We propose that overexpression of *KLU* activates cytokinin signaling and proline metabolism, thus prolonging leaf longevity and enhancing drought-stress tolerance (Figure 6).

Biochemically, KLU, belonging to the CYP450 family, has been predicted to produce a novel growth regulator that differs from the characterized phytohormones (Anastasiou et al., 2007). KLU has tremendous effects on plant growth and development, including in leaf initiation, flowering time, apical dominance, inflorescence organ, fruit size, and megagametogenesis (Miyoshi et al., 2004; Eriksson et al., 2010). Recently, a tandem duplication of the tomato *KLU* gene, *SlKLUH*, was shown to be the cause of a quantitative trait loci (QTL) of fruit weight (Alonge et al., 2020). Arabidopsis *KLU* is highly expressed in the shoot apical meristem, and exhibits quite low expression in the leaves (Zondlo and Irish, 1999). Our transcriptome sequencing analysis also detected modest expression of *KLU* in WT (Supplemental Figure 1, D). Leaf senescence normally occurring in the later reproductive stage is strongly linked with plant developmental processes (Koyama, 2018). The acceleration of leaf initiation in the *klu* mutant might advance the whole development process. Taking this into account, we could not rule out the possibility that early leaf senescence observed in the *klu* mutant was partially due to developmental disorders caused by the disruption of *KLU*.

Phytohormones play important roles in leaf senescence (Goldthwaite, 1987). Cytokinin is one of the well-characterized phytohormones involved in leaf senescence. Either high levels of cytokinin contents or increased ARR2 phosphorylation mediated by cytokinin receptor AHK3 effectively delay leaf senescence (Kim et al., 2006). Transcriptome sequencing analysis showed that *CKX1* and *CKX4*, the key enzymes responsible for cytokinin catabolism, were decreased in *KLU*-overexpressing plants (Figure 3). Consistently, phytohormone measurements showed that cytokinin was highly accumulated in *KLU*-overexpressing plants, whereas it was slightly decreased in the *klu* mutant (Figure 2). It is unlikely that KLU functions in the cytokinin biosynthetic pathways because CYP735A has been characterized as the enzyme for the conversion of IP-type cytokinin to *t*Z-type cytokinin (Takei et al., 2004). No detectable *t*Z-type cytokinins were found in the loss-of-function *CYP735A* mutant, suggesting that CYP735A has no redundant functions with other enzymes in cytokinin biosynthesis (Kiba et al., 2013). Strikingly, in addition to the increased level of cytokinin in *35S:KLU* plants, several type-A *ARR* genes were decreased. Type-A ARRs negatively regulate cytokinin signaling by competing with type-B ARRs for phosphoryl groups. The decreased expression of type-A *ARR* might contribute to activation of the cytokinin signaling pathway. Generally, type-A *ARR* genes are induced by cytokinins. The discrepancy between the increased cytokinin level and down-regulation of type-A *ARR* genes implies that KLU uncouples cytokinin perception from its signaling pathway via unknown mechanisms. Our metabolome data showed that the metabolite profiles of *ckx1* and *ckx4* were distinct from that of *KLU*-overexpressing plants (Figure 5), supporting the idea that the phenotypic changes associated with *KLU* overexpression are not directly caused by an increased level of *t*Z. Taken together, our data indicate that KLU activates cytokinin signaling by coordinately controlling cytokinin hemostasis and cytokinin-responsive regulators. In addition to leaf senescence, cytokinins play critical roles in plant growth and development (Kieber and Schaller, 2018). In fact, several phenotypic features observed in *KLU*-overexpressing plants appear to be related with the biological functions of cytokinin signaling. On the one hand, the enlarged seed size of *KLU*-overexpressing plants, consistent with elevated levels of cytokinin, correlates with increased meristem size (Su et al., 2011). On the other hand, the increased number of shoot branches in the *klu* mutant (Supplemental Figure S5) is consistent with decreased apical dominance in those mutants with reduced *t*Z-type cytokinins (Müller and Leyser, 2011). Moreover, in accordance with the fact that increased levels of cytokinin has negative effects on nitrogen assimilation (Kiba et al., 2011), we observed altered expression of genes involved in nitrate transport, nitrogen assimilation, and proline metabolism in the *KLU*-overexpressing lines, as well as the accumulation of proline. Besides cytokinin, auxin has a pivotal role in plant growth and development. However, since IAA accumulated in both *klu* and *KLU*-overexpressing plants (Figure 2), IAA is unlikely the cause of the different leaf senescence phenotypes of *klu* and *KLU*-overexpression plants. By contrast, some auxin biosynthetic genes, such as *CYP79B2*, displayed opposite expression patterns between *klu* and *KLU*-overexpressing plants (Supplemental Figure S4). Given that several auxin biosynthetic genes, including *CYP79B2*, are responsive to cytokinin (Jones et al., 2010), IAA biosynthesis might be partially affected by altered levels of cytokinin in the *klu* mutant and *KLU*-overexpressing plants.

*KLU*-overexpressing plants showed enhanced drought-stress tolerance, decreased water loss, and greater stomatal closure (Figure 6 and Supplemental Figure S14), whereas accumulation of the key phytohormone in drought-stress responses, ABA, was reduced in both *klu* and *KLU*-overexpressing plants (Figure 2). The decreased levels of ABA appeared to be consistent with the decreased expression of the ABA biosynthetic genes *NINE-CIS-EPOXYCAROTENOID DIOXYGENASE* 2 (*NCED2*) and *NCED3* and the unchanged expression of the ABA-responsive genes *ABA-RESPONSIVE ELEMENT BINDING PROTEIN 2* (*AREB2*) and *RESPONSIVE TO DESICCATION 29B* (*RD29B*) in both *klu* and *KLU*-overexpressing lines (Supplemental Figure S4). By contrast, the expression of an ATP-binding cassette (ABC) transporter of ABA, *ABCG40*, was significantly increased in *KLU*-overexpressing plants, implying that the ABCG40-mediated ABA import in guard cells was enhanced. Collectively, these data show that the overexpression of *KLU* might affect local ABA transport without altering the general enhancement of ABA responses, likely consistent with the previous report that the overproduction of cytokinin results in delayed leaf senescence and increased drought-stress tolerance without altered ABA contents (Rivero et al., 2007). Notably, the expression of type-A ARRs, such as *ARR5* and *ARR7*, was shown to be decreased in cytokinin-deficient lines that displayed enhanced drought- and salt-stress tolerance (Nishiyama et al., 2011), as in the *KLU*-overexpressing lines (Figure 3). Although the role of type-A ARRs in stress signaling pathways is under discussion because of their functional redundancy and complexity (Wohlbach et al., 2008), cold-inducible type-A ARRs, including *ARR7*, appear to have a negative regulatory role in freezing tolerance (Jeon et al., 2010). In accordance with the elevated SA level, the biosynthetic gene, *ISOCHORISMATE SYNTHASE 1* (*ICS1*), and the responsive gene, *PATHOGENESIS-RELATED 1* (*PR1*), showed increased expression levels in *KLU*-overexpressing plants (Supplemental Figure S4). In contrast to the inhibitory effect of cytokinin on ABA-induced stomatal closure (Tanaka et al., 2006), cytokinin has been reported to stimulate SA biosynthesis and signaling (Choi et al., 2010) and SA-mediated stomatal closure (Arnaud et al., 2017). Therefore, the increased levels of cytokinin and SA in *KLU*-overexpressing plants might also reduce water loss by promoting stomatal closure. More detailed phytohormone interactions downstream of KLU will be revealed by further analyses of lines with knockout mutation of multiple *KLU* family genes, thus overcoming their functional redundancy (Supplemental Figure S1).

Integration of metabolic analysis and transcriptomic analysis enables us to gain insight into the connections and relationships of different biological layers. Whereas nitrate uptake and efflux are mediated by the nitrate transporters expressed in roots, several nitrate transporters, including *NRT1.4*, *NRT1.11*, and *NRT1.12*, are expressed in shoots. The leaf petiole is a storage site of nitrate, and NRT1.4 expressed in the leaf petiole is essential for its storage (Chiu et al., 2004). NRT1.11 and NRT1.12 expressed in the companion cells of the major veins are involved in xylem-to-phloem transfer of nitrate for redistributing nitrate into developing leaves (Hsu and Tsay, 2013). The decreased expression of these nitrate transporter genes and glutamine synthetase (GS or GLN) genes, such as *GLN1;4*, in the *KLU*-overexpressing lines (Supplemental Figure S13) might impair nitrate storage and allocation and nitrogen assimilation for proper growth and development. Given that the levels of arginine and ornithine, which accumulated in the shoot under nitrogen-limited conditions (Tschoep et al., 2009), were increased in the *KLU*-overexpressing lines (Figure 5), the overexpression of *KLU* might partially cause nitrogen deficiency. Moreover, given that *GLUTAMATE DEHYDROGENASE 2* (*GDH2*) was shown to be induced in response to dark conditions (Miyashita and Good, 2008), similar to as in the *KLU*-overexpressing lines compared with the *klu* mutant (Supplemental Figure S13), these transcriptional and metabolic changes might be indicative of the carbon- and nitrogen-limited status of *KLU*-overexpressing plants. Since leaf senescence is affected by both endogenous factors, such as age and phytohormones, and environmental signals, such as darkness and nutrient supply, the transcriptional and metabolic changes seemed to be contradictory with the delayed leaf-senescence phenotypes in *KLU*-overexpressing plants (Figure 4). This contradiction might be explained by increased proline levels accompanied by the up-regulation of its metabolic genes (Figure 5 and Supplemental Figure S13). As a stress-induced protectant, proline has unique biological roles in protecting plants against many abiotic stresses (Szabados and Savouré, 2010). More importantly, proline metabolism is involved in ATP synthesis, maintenance of NADP^+^/NADPH balance, and ROS production (Liang et al., 2013). Indeed, proline dehydrogenases (ProDHs) in mitochondria were recently shown to serve in the generation of glutamate and energy by proline oxidation during dark-induced leaf senescence (Launay et al., 2019), likely consistent with decreased levels of proline during developmental leaf senescence (Chrobok et al., 2016), especially in the base region of leaves during vegetative stages (Watanabe et al., 2013). Enhanced proline biosynthesis and catabolism might prevent dark-induced senescence in *KLU*-overexpressing plants (Figure 6). Proline accumulation in response to low water potential is partially regulated by ABA (Sharma and Verslues, 2010), whereas proline accumulation is impaired in cytokinin-receptor mutants (Kumar and Verslues, 2015). Thus, the increased proline contents in *KLU*-overexpressing plants might be caused partly by the increased level of *t*Z (Figure 2). Because proline level was not changed in *ckx1* or *ckx4* (Figure 5), the *KLU* overexpression appeared to affect other factors related with proline accumulation, such as ABA transport. Collectively, proline catabolism, a machinery for nutrient recycling and remobilization during leaf senescence, might be activated as a consequence of proline accumulation by the increased level of *t*Z in *KLU*-overexpressing plants, leading to extended leaf longevity. Our work illustrates the relationship between cytokinin signaling and proline metabolism and provides experimental evidence for the potential role of proline in leaf senescence.

## Conclusion

In this study, we characterized a function of *KLU* as a negative regulator of leaf senescence. The integration of transcriptome sequencing analysis and metabolite profiling revealed that *KLU* overexpression activates cytokinin signaling by coordinately repressing cytokinin catabolism genes and the negative cytokinin response regulatory genes. Consequently, *KLU*-overexpression plants showed delayed leaf senescence. Moreover, we found that both activation of the cytokinin signaling pathway and *KLU* overexpression accelerate proline metabolism. Considering the negative regulation of leaf senescence by KLU and its positive effects on plant fitness, manipulation of *KLU* expression levels may be a powerful strategy to improve agricultural plant productivity.

## Materials and methods

### Plant materials and growth conditions

Arabidopsis (*Arabidopsis thaliana* ecotype Columbia [Col-0]) was used as the WT in this study. The *klu* mutant (SALK_024697C) used in this study is the Col-0 background (Zhao et al., 2018). The T-DNA insertion mutants *ckx1-1* (SALK_204043C) and *ckx4-1* (SALK_055204C; Bartrina et al., 2011) were provided by the Nottingham Arabidopsis Stock Centre. Seeds were surface-sterilized and plated on Murashige and Skoog (MS) medium (4.3 g/L MS salts, 1% [w/v] sucrose, pH 5.7–5.8, and 8 g/L agar). After stratification at 4°C for 2 d, the plates were move to a growth cabinet with 16-h light/8-h dark cycles for 5–7 d. The seedlings were transferred to soil and grown at 22°C in a phytotron under an 8-h light (PAR of 120–150 µE m^−2^ s^−1^)/16-h dark cycle unless otherwise noted. For drought tolerance experiments, plant were grown under the same phytotron conditions except that watering was withheld. For the dark extension treatment, plants were grown under the same phytotron conditions except that they were covered with a black box.

### Construction of plasmids and generation of transgenic plants

To construct the *35S:KLU-GFP* plasmid, the full-length coding sequences of *KLU* were amplified using primers KLUGWF and KLUGWR, then was cloned into the *pDONR-207* vector (Invitrogen) and introduced into the plant binary vector *pK7FWG2* by LR reaction (Karimi et al., 2002). DNA constructs were verified by DNA sequencing analysis and were electroporated into *Agrobacterium tumefaciens* GV3101, which was used to transform Col-0 plants by the floral dip method (Clough and Bent, 1998). Successfully transformed T1 plants were selected on MS medium containing with 50 mg/L kanamycin.

### Assays of leaf senescence and chlorophyll measurement

Age-dependent leaf senescence was evaluated as reported previously (Woo et al., 2001). The leaf senescence photograph and chlorophyll measurement were carried out using the third rosette leaf at each time point as indicated in the figures. Chlorophyll contents of leaves were measured as described previously (Richardson et al., 2002). The photochemical efficiency of photosystem II was measured by the Imaging-PAMs (Heinz Walz GmbH, Germany) following the user instructions. All differences between the group were analyzed by Student’s *t* test.

### RNA isolation, transcriptome sequencing, and reverse transcription quantitative PCR

Total RNA was extracted from aerial organs of individual plants using TRIzol reagent (Invitrogen) according to the manufacturer’s instructions. Total RNA (2.5 μg) was treated with DNase I and used for complementary DNA synthesis with Thermo Scientific Maxima First Strand cDNA Synthesis Kit. qPCR experiments were performed with gene-specific primers (Supplemental Table S12) in the reaction system of a Power SYBR Green PCR Master Mix kit (Applied Biosystems) on ABI PRISM 7900HT sequence detection system (Applied Biosystems). The Arabidopsis *UBIQUITIN 5* or *ACTIN 2* genes were used as internal controls. For transcriptome sequencing, total RNA was extracted from the whole rosette of three individual plants of each genotype at 35 DAG by Zymo-Spin IIC RNA column (Zymo Research Europe GmbH). The isolated total RNA was assessed for quality by Agilent Bioanalyzer. For each sample, 500 ng was used as input for polyA enrichment followed by fragmentation and library preparation as recommended by the vendor (NEBNext Ultra II Directional RNA Library Prep Kit Illumina). Quality and size distribution of libraries were again inspected (Agilent Tapestation). Paired end 2 × 150-bp read-based sequencing was performed on an Illumina HiSeq3000. The RNA-seq raw reads were mapped to the *A. thaliana* genome (build TAIR 10).

### Identification of DEGs, and GO and KEGG enrichment analysis

Differential expression analyses were performed with DESeq2 version 1.36 (Love et al., 2014) in the R environment. The genes with adjusted *P*-value less than 0.05 were regarded as DEGs. For the GO and KEGG enrichment analysis, we further filtered the DEGs by choosing the common genes found in both *klu* versus *35S:KLU1* and *klu* versus *35S:KLU2*, named as *klu* versus OE*.* Similarly, the Col-0 versus OE are the DEGs that share both Col-0 versus *35S:KLU1* and Col-0 versus *35S:KLU2.* GO and KEGG pathway enrichment analysis was performed using R package clusterProfiler version 3.10 (Yu et al., 2012).

### Metabolite measurement by gas chromatography–mass spectrometry

The whole rosettes of five or six biological replicates for each genotype were quickly cut and stored in liquid nitrogen. Metabolite profiling of these samples was carried out by gas chromatography–mass spectrometry (ChromaTOF software, Pegasus driver 1.61; LECO) as described previously (Lisec et al., 2006). In brief, homogenized samples (∼50 mg) were extracted in 1,400 μL of methanol. Subsequently, 60 µL of internal standard (0.2 mg ribitol mL^−1^ water) was added as a quantification standard. The extraction, derivatization, standard addition, and sample injection were conducted as described in Lisec et al. (2006). An autosampler Gerstel Multi-Purpose system (Gerstel GmbH & Co.KG, Mülheim an der Ruhr, Germany) used to inject the samples to a chromatograph coupled to a time-of-flight mass spectrometer (GC–MS) system (Leco Pegasus HT TOF-MS [LECO Corporation, St. Joseph, MI, USA]). The chromatograms and mass spectra were evaluated using TagFinder 4.0 software (Luedemann et al., 2012). Metabolite identification was manually checked by both the mass spectral and retention index collection of the Golm Metabolome Database (Kopka et al., 2005). The relative content of metabolites was calculated by normalization of signal intensity to that of ribitol, which was added as an internal standard, and by the fresh weight of the material. All data were also processed using the Xcalibur 4.0 software (Thermo Fisher Scientific, Waltham, MA, USA) to verify the metabolite identification and annotation. Identification and annotation of detected peaks followed the recommendations for reporting metabolite data (Supplemental DataSet S1; Fernie et al., 2011). Statistical analyses were performed on MetaboAnalyst 4.0 (https://www.metaboanalyst.ca/; Chong et al., 2019).

### Hormone measurement and exogenous *trans*-zeatin treatment

Cytokinins (*trans*-zeatin), gibberellins (GA1, GA4, and GA3), indole-3-acetic acid, ABA, SA, jasmonic acid, and the ethylene precursor 1-aminocyclopropane-1-carboxylic acid (ACC) were analyzed in mature leaves according to Albacete et al. (2008) with some modifications (Albacete et al., 2008). Ten microliters of extracted sample was injected in a Ultra high performance liquid chromatography (UHPLC)–MS system consisting of an Accela Series U-HPLC (ThermoFisher Scientific, Waltham, MA, USA) coupled to an Exactive mass spectrometer (ThermoFisher Scientific, Waltham, MA, USA) using a heated electrospray ionization (HESI) interface. Mass spectra were obtained using the Xcalibur software version 2.2 (ThermoFisher Scientific, Waltham, MA, USA). For quantification of the phytohormones, calibration curves were constructed for each analyzed component (1, 10, 50, and 100 μg/L) and corrected for 10 μg/L deuterated internal standards. Recovery percentages ranged between 92% and 95%. To examine responses to *trans*-zeatin treatment, the seeds of each genotype were surface-sterilized and plated on vertical MS plate (10 g/L agar) supplemented with indicated concentration of *trans*-zeatin.

### Stomatal aperture measurement

Stomatal apertures were examined as described in Fujita et al. (2009) with minor modifications. Fully expanded leaves of 6-week-old Arabidopsis plants grown in soil were detached and floated on stomatal opening buffer containing 20-mM KCl, 1-mM CaCl_2_, and 5-mM 2-(*N*-morpholino)ethanesulfonic acid (MES; pH 6.15, potassium hydroxide [KOH]; Pei et al., 1998) for 2 h to preopen the stomata. Subsequently, ABA or ethanol (solvent control) was added to the opening buffer to a final concentration of 5- or 10-µM ABA. After 3 h of incubation, images of the abaxial epidermis were taken as described in Medeiros et al. (2017).

## Accession numbers

The raw RNA-seq data reported in this article have been deposited in Gene Expression Omnibus (Barrett et al., 2013), under accession number GSE128655. Further sequence data from this article can be found in the GenBank/EMBL data libraries under accession numbers: *KLU* (AT1G13710), *CKX1* (AT2G41510), *CKX4* (AT4G29740), and *SAG12* (AT5G45890).

## Supplemental data

**Supplemental Figure S1**. Overview of the transcriptome changes and the expression of CYP78A5 family genes.

**Supplemental Figure S2**. GO enrichment analysis.

**Supplemental Figure S3**. The expression of the genes associated with phytohormone metabolism, transport, perception, and signaling in *klu* and *35S:KLU* plants analyzed by RNA-sequencing.

**Supplemental Figure S4**. The expression of the selected genes associated with phytohormone metabolism, transport, perception, and signaling in *klu* and *35S:KLU* plants analyzed by RT-qPCR.

**Supplemental Figure S5**. Phenotypes of *klu* and *KLU*-overexpression plants.

**Supplemental Figure S6**. Growth characteristics of the *ckx* mutants.

**Supplemental Figure S7**. Top metabolites contributing the PCA plots.

**Supplemental Figure S8**. The metabolites showed constitutive increased accumulation in the *KLU*-overexpressing lines.

**Supplemental Figure S9**. The proteinogenic amino acids more accumulated in the *KLU*-overexpressing lines than in the *klu* mutant.

**Supplemental Figure S10**. The non-proteinogenic amino acids more accumulated in the *KLU*-overexpressing lines than in the *klu* mutant.

**Supplemental Figure S11**. The organic acids more accumulated in the *KLU*-overexpressing lines than in the *klu* mutant.

**Supplemental Figure S12**. The levels of sugars and sugar alcohols were altered in the *KLU*-overexpressing lines compared to in the *klu* mutant.

**Supplemental Figure S13**. The expression of the genes associated with nitrogen assimilation and proline metabolism in *klu* and *35S:KLU* plants analyzed by RNA-sequencing.

**Supplemental Figure S14**. Stomatal closure of guard cells in response to ABA.

**Supplemental Table S1**. List of genes with RPKM and *P*-value in this RNA-seq data

**Supplemental Table S2**. Expression of the genes associated with cytokinin metabolism, transport, perception, and signaling in *klu* and *35S:KLU* plants

**Supplemental Table S3**. Expression of the genes associated with auxin metabolism, transport, perception, and signaling in *klu* and *35S:KLU* plants

**Supplemental Table S4**. Expression of the genes associated with GA metabolism, transport, perception, and signaling in *klu* and *35S:KLU* plants

**Supplemental Table S5**. Expression of the genes associated with SA metabolism, transport, perception, and signaling in *klu* and *35S:KLU* plants

**Supplemental Table S6**. Expression of the genes associated with ABA metabolism, transport, perception, and signaling in *klu* and *35S:KLU* plants

**Supplemental Table S7**. Expression of the genes associated with JA metabolism, transport, perception, and signaling in *klu* and *35S:KLU* plants

**Supplemental Table S8**. Expression of the genes associated with BR metabolism, transport, perception, and signaling in *klu* and *35S:KLU* plants

**Supplemental Table S9**. Expression of the genes associated with ethylene metabolism, transport, perception, and signaling in *klu* and *35S:KLU* plants

**Supplemental Table S10**. Expression of the genes associated with nitrogen assimilation, remobilization, and transport in *klu* and *35S:KLU* plants.

**Supplemental Table S11**. Expression of the genes associated with proline metabolism in *klu* and *35S:KLU* plants

**Supplemental Table S12**. Primers used in this study

**Supplemental Dataset S1**. Metabolite data report for the compounds analyzed in this study.

## Supplementary Material

kiaa034_Supplementary_Data

## Notes

L.J. and A.R.F. conceived the research plans. A.R.F. supervised the experiments. L.J., T.Y., and S.S. performed the experiments. L.J., T.Y., and Y.J. analyzed the data. S.A. and F.P.-A. provided the technical assistance. L.J., T.Y., and A.R.F. analyzed the data and wrote the manuscript.

The author responsible for distribution of materials integral to the findings presented in this article in accordance with the policy described in the Instructions for Authors (https://academic.oup.com/plphys) is: Alisdair R. Fernie (Fernie@mpimp-golm.mpg.de).

## References

[kiaa034-B1] Adamski NM , Anastasiou E, Eriksson S, O’Neill CM, Lenhard M (2009) Local maternal control of seed size by KLUH/CYP78A5-dependent growth signaling. Proc Natl Acad Sci U S A 106: 20115–20120 19892740 10.1073/pnas.0907024106 PMC2785301

[kiaa034-B2] Agüera E , Cabello P, de la Haba P (2010) Induction of leaf senescence by low nitrogen nutrition in sunflower (*Helianthus annuus*) plants. Physiol Plant 138: 256–267 20051027 10.1111/j.1399-3054.2009.01336.x

[kiaa034-B3] Albacete A , Ghanem ME, Martínez-Andújar C, Acosta M, Sánchez-Bravo J, Martínez V, Lutts S, Dodd IC, Pérez-Alfocea F (2008) Hormonal changes in relation to biomass partitioning and shoot growth impairment in salinized tomato (*Solanum lycopersicum* L.) plants. J Exp Bot 59: 4119–4131 19036841 10.1093/jxb/ern251 PMC2639025

[kiaa034-B4] Alonge M , Wang X, Benoit M, Soyk S, Pereira L, Zhang L, Suresh H, Ramakrishnan S, Maumus F, Ciren D, et al (2020) Major impacts of widespread structural variation on gene expression and crop improvement in tomato. Cell 182: 145–161.e123 32553272 10.1016/j.cell.2020.05.021 PMC7354227

[kiaa034-B5] Anastasiou E , Kenz S, Gerstung M, MacLean D, Timmer J, Fleck C, Lenhard M (2007) Control of plant organ size by KLUH/CYP78A5-dependent intercellular signaling. Dev Cell 13: 843–856 18061566 10.1016/j.devcel.2007.10.001

[kiaa034-B7] Arnaud D , Lee S, Takebayashi Y, Choi D, Choi J, Sakakibara H, Hwang I (2017) Cytokinin-mediated regulation of reactive oxygen species homeostasis modulates stomatal immunity in Arabidopsis. Plant Cell 29: 543–559 28254779 10.1105/tpc.16.00583 PMC5385949

[kiaa034-B8] Bak S , Beisson F, Bishop G, Hamberger B, Höfer R, Paquette S, Werck-Reichhart D (2011) Cytochromes p450. Arabidopsis Book 9: e0144 22303269 10.1199/tab.0144 PMC3268508

[kiaa034-B9] Balibrea Lara ME , Gonzalez Garcia MC, Fatima T, Ehness R, Lee TK, Proels R, Tanner W, Roitsch T (2004) Extracellular invertase is an essential component of cytokinin-mediated delay of senescence. Plant Cell 16: 1276–1287 15100396 10.1105/tpc.018929 PMC423215

[kiaa034-B10] Barrett T , Wilhite SE, Ledoux P, Evangelista C, Kim IF, Tomashevsky M, Marshall KA, Phillippy KH, Sherman PM, Holko M, et al (2013) NCBI GEO: archive for functional genomics data sets—update. Nucleic Acids Res 41: D991–995 23193258 10.1093/nar/gks1193 PMC3531084

[kiaa034-B11] Bartrina I , Otto E, Strnad M, Werner T, Schmülling T (2011) Cytokinin regulates the activity of reproductive meristems, flower organ size, ovule formation, and thus seed yield in *Arabidopsis thaliana*. Plant Cell 23: 69–80 21224426 10.1105/tpc.110.079079 PMC3051259

[kiaa034-B12] Becker W , Apel K (1993) Differences in gene-expression between natural and artificially induced leaf senescence. Planta 189: 74–79

[kiaa034-B13] Bleecker AB (1998) The evolutionary basis of leaf senescence: method to the madness? Curr Opin Plant Biol 1: 73–78 10066560 10.1016/s1369-5266(98)80131-3

[kiaa034-B14] Brandstatter I , Kieber JJ (1998) Two genes with similarity to bacterial response regulators are rapidly and specifically induced by cytokinin in Arabidopsis. Plant Cell 10: 1009–1019 9634588 10.1105/tpc.10.6.1009 PMC144033

[kiaa034-B15] Brzobohatý B , Moore I, Kristoffersen P, Bako L, Campos N, Schell J, Palme K (1993) Release of active cytokinin by a beta-glucosidase localized to the maize root meristem. Science 262: 1051–1054 8235622 10.1126/science.8235622

[kiaa034-B16] Buchanan-Wollaston V , Page T, Harrison E, Breeze E, Lim PO, Nam HG, Lin JF, Wu SH, Swidzinski J, Ishizaki K, et al (2005) Comparative transcriptome analysis reveals significant differences in gene expression and signalling pathways between developmental and dark/starvation-induced senescence in Arabidopsis. Plant J 42: 567–585 15860015 10.1111/j.1365-313X.2005.02399.x

[kiaa034-B17] Calderini O , Bovone T, Scotti C, Pupilli F, Piano E, Arcioni S (2007) Delay of leaf senescence in *Medicago sativa* transformed with the ipt gene controlled by the senescence-specific promoter SAG12. Plant Cell Rep 26: 611–615 17149639 10.1007/s00299-006-0262-y

[kiaa034-B18] Chakrabarti M , Zhang N, Sauvage C, Muños S, Blanca J, Cañizares J, Diez MJ, Schneider R, Mazourek M, McClead J et al (2013) A cytochrome P450 regulates a domestication trait in cultivated tomato. Proc Natl Acad Sci U S A 110: 17125–17130 24082112 10.1073/pnas.1307313110 PMC3801035

[kiaa034-B19] Chiu CC , Lin CS, Hsia AP, Su RC, Lin HL, Tsay YF (2004) Mutation of a nitrate transporter, AtNRT1:4, results in a reduced petiole nitrate content and altered leaf development. Plant Cell Physiol 45: 1139–1148 15509836 10.1093/pcp/pch143

[kiaa034-B20] Choi J , Huh SU, Kojima M, Sakakibara H, Paek KH, Hwang I (2010) The cytokinin-activated transcription factor ARR2 promotes plant immunity via TGA3/NPR1-dependent salicylic acid signaling in Arabidopsis. Dev Cell 19: 284–295 20708590 10.1016/j.devcel.2010.07.011

[kiaa034-B21] Chong J , Wishart DS, Xia J (2019) Using MetaboAnalyst 4.0 for comprehensive and integrative metabolomics data analysis. Curr Protoc Bioinformatics 68: e86 31756036 10.1002/cpbi.86

[kiaa034-B22] Chrobok D , Law SR, Brouwer B, Lindén P, Ziolkowska A, Liebsch D, Narsai R, Szal B, Moritz T, Rouhier N et al (2016) Dissecting the metabolic role of mitochondria during developmental leaf senescence. Plant Physiol 172: 2132–2153 27744300 10.1104/pp.16.01463 PMC5129728

[kiaa034-B23] Clough SJ , Bent AF (1998) Floral dip: a simplified method for Agrobacterium-mediated transformation of *Arabidopsis thaliana*. Plant J 16: 735–743 10069079 10.1046/j.1365-313x.1998.00343.x

[kiaa034-B24] D’Agostino IB , Deruère J, Kieber JJ (2000) Characterization of the response of the Arabidopsis response regulator gene family to cytokinin. Plant Physiol 124: 1706–1717 11115887 10.1104/pp.124.4.1706 PMC59868

[kiaa034-B25] Diaz C , Lemaître T, Christ A, Azzopardi M, Kato Y, Sato F, Morot-Gaudry JF, Le Dily F, Masclaux-Daubresse C (2008) Nitrogen recycling and remobilization are differentially controlled by leaf senescence and development stage in Arabidopsis under low nitrogen nutrition. Plant Physiol 147: 1437–1449 18467460 10.1104/pp.108.119040 PMC2442554

[kiaa034-B26] Dyer TA , Osborne DJ (1971) Leaf nucleic acids .2. Metabolism during senescence and effect of kinetin. J Exp Bot 22: 552–560

[kiaa034-B27] El-Showk S , Ruonala R, Helariutta Y (2013) Crossing paths: cytokinin signalling and crosstalk. Development 140: 1373–1383 23482484 10.1242/dev.086371

[kiaa034-B28] Eriksson S , Stransfeld L, Adamski NM, Breuninger H, Lenhard M (2010) KLUH/CYP78A5-dependent growth signaling coordinates floral organ growth in Arabidopsis. Curr Biol 20: 527–532 20188559 10.1016/j.cub.2010.01.039

[kiaa034-B29] Fang WJ , Wang ZB, Cui RF, Li J, Li YH (2012) Maternal control of seed size by EOD3/CYP78A6 in *Arabidopsis thaliana*. Plant J 70: 929–939 22251317 10.1111/j.1365-313X.2012.04907.x

[kiaa034-B30] Fernie AR , Aharoni A, Willmitzer L, Stitt M, Tohge T, Kopka J, Carroll AJ, Saito K, Fraser PD, DeLuca V (2011) Recommendations for reporting metabolite data. Plant Cell 23: 2477–2482 21771932 10.1105/tpc.111.086272 PMC3226225

[kiaa034-B31] Fujita Y , Nakashima K, Yoshida T, Katagiri T, Kidokoro S, Kanamori N, Umezawa T, Fujita M, Maruyama K, Ishiyama K et al (2009) Three SnRK2 protein kinases are the main positive regulators of abscisic acid signaling in response to water stress in Arabidopsis. Plant Cell Physiol 50: 2123–2132 19880399 10.1093/pcp/pcp147

[kiaa034-B32] Gan S , Amasino RM (1995) Inhibition of leaf senescence by autoregulated production of cytokinin. Science 270: 1986–1988 8592746 10.1126/science.270.5244.1986

[kiaa034-B33] Gan SS , Amasino RM (1996) Cytokinins in plant senescence: From spray and pray to clone and play. Bioessays 18: 557–565

[kiaa034-B34] Goldthwaite JJ (1987) Hormones in plant senescence. In Davies PJ, ed., Plant Hormones and Their Role in Plant Growth and Development. Springer, Netherlands, Dordrecht, pp. 553–573

[kiaa034-B35] Guo Y (2013) Towards systems biological understanding of leaf senescence. Plant Mol Biol 82: 519–528 23065109 10.1007/s11103-012-9974-2

[kiaa034-B36] Guo Y , Gan S (2005) Leaf senescence: signals, execution, and regulation. Curr Top Dev Biol 71: 83–112 16344103 10.1016/S0070-2153(05)71003-6

[kiaa034-B37] Havé M , Marmagne A, Chardon F, Masclaux-Daubresse C (2017) Nitrogen remobilization during leaf senescence: lessons from Arabidopsis to crops. J Exp Bot 68: 2513–2529 27707774 10.1093/jxb/erw365

[kiaa034-B40] Hsu PK , Tsay YF (2013) Two phloem nitrate transporters, NRT1.11 and NRT1.12, are important for redistributing xylem-borne nitrate to enhance plant growth. Plant Physiol 163: 844–856 24006285 10.1104/pp.113.226563 PMC3793062

[kiaa034-B41] Hu Y , Jiang Y, Han X, Wang H, Pan J, Yu D (2017) Jasmonate regulates leaf senescence and tolerance to cold stress: crosstalk with other phytohormones. J Exp Bot 68: 1361–1369 28201612 10.1093/jxb/erx004

[kiaa034-B42] Hutchison CE , Kieber JJ (2002) Cytokinin signaling in Arabidopsis. Plant Cell 14: S47–S59 12045269 10.1105/tpc.010444 PMC151247

[kiaa034-B43] Hwang I , Sheen J (2001) Two-component circuitry in Arabidopsis cytokinin signal transduction. Nature 413: 383–389 11574878 10.1038/35096500

[kiaa034-B44] Hwang I , Sheen J, Müller B (2012) Cytokinin signaling networks. Annu Rev Plant Biol 63: 353–380 22554243 10.1146/annurev-arplant-042811-105503

[kiaa034-B45] Jeon J , Kim NY, Kim S, Kang NY, Novák O, Ku SJ, Cho C, Lee DJ, Lee EJ, Strnad M et al (2010) A subset of cytokinin two-component signaling system plays a role in cold temperature stress response in Arabidopsis. J Biol Chem 285: 23371–23386 20463025 10.1074/jbc.M109.096644 PMC2906329

[kiaa034-B46] Jones B , Gunnerås SA, Petersson SV, Tarkowski P, Graham N, May S, Dolezal K, Sandberg G, Ljung K (2010) Cytokinin regulation of auxin synthesis in Arabidopsis involves a homeostatic feedback loop regulated via auxin and cytokinin signal transduction. Plant Cell 22: 2956–2969 20823193 10.1105/tpc.110.074856 PMC2965550

[kiaa034-B47] Kakimoto T (2001) Identification of plant cytokinin biosynthetic enzymes as dimethylallyl diphosphate: ATP/ADP isopentenyltransferases. Plant Cell Physiol 42: 677–685 11479373 10.1093/pcp/pce112

[kiaa034-B48] Karimi M , Inzé D, Depicker A (2002) GATEWAY vectors for Agrobacterium-mediated plant transformation. Trends Plant Sci 7: 193–195 11992820 10.1016/s1360-1385(02)02251-3

[kiaa034-B49] Kazama T , Ichihashi Y, Murata S, Tsukaya H (2010) The mechanism of cell cycle arrest front progression explained by a KLUH/CYP78A5-dependent mobile growth factor in developing leaves of *Arabidopsis thaliana*. Plant Cell Physiol 51: 1046–1054 20395288 10.1093/pcp/pcq051

[kiaa034-B50] Khan M , Rozhon W, Poppenberger B (2014) The role of hormones in the aging of plants—a mini-review. Gerontology 60: 49–55 24135638 10.1159/000354334

[kiaa034-B51] Kiba T , Kudo T, Kojima M, Sakakibara H (2011) Hormonal control of nitrogen acquisition: roles of auxin, abscisic acid, and cytokinin. J Exp Bot 62: 1399–1409 21196475 10.1093/jxb/erq410

[kiaa034-B52] Kiba T , Takei K, Kojima M, Sakakibara H (2013) Side-chain modification of cytokinins controls shoot growth in Arabidopsis. Dev Cell 27: 452–461 24286826 10.1016/j.devcel.2013.10.004

[kiaa034-B53] Kieber JJ , Schaller GE (2014) Cytokinins. Arabidopsis Book 12: e0168 24465173 10.1199/tab.0168 PMC3894907

[kiaa034-B54] Kieber JJ , Schaller GE (2018) Cytokinin signaling in plant development. Development 145: dev149344 29487105 10.1242/dev.149344

[kiaa034-B55] Kim HJ , Ryu H, Hong SH, Woo HR, Lim PO, Lee IC, Sheen J, Nam HG, Hwang I (2006) Cytokinin-mediated control of leaf longevity by AHK3 through phosphorylation of ARR2 in Arabidopsis. Proc Natl Acad Sci U S A 103: 814–819 16407152 10.1073/pnas.0505150103 PMC1334631

[kiaa034-B56] Kim J , Woo HR, Nam HG (2016) Toward systems understanding of leaf senescence: an integrated multi-omics perspective on leaf senescence research. Mol Plant 9: 813–825 27174403 10.1016/j.molp.2016.04.017

[kiaa034-B57] Kopka J , Schauer N, Krueger S, Birkemeyer C, Usadel B, Bergmüller E, Dörmann P, Weckwerth W, Gibon Y, Stitt M et al (2005) GMD@CSB.DB: the Golm Metabolome Database. Bioinformatics 21: 1635–1638 15613389 10.1093/bioinformatics/bti236

[kiaa034-B58] Koyama T (2014) The roles of ethylene and transcription factors in the regulation of onset of leaf senescence. Front Plant Sci 5: 650 25505475 10.3389/fpls.2014.00650 PMC4243489

[kiaa034-B59] Koyama T (2018) A hidden link between leaf development and senescence. Plant Sci 276: 105–110 30348308 10.1016/j.plantsci.2018.08.006

[kiaa034-B60] Kumar MN , Verslues PE (2015) Stress physiology functions of the Arabidopsis histidine kinase cytokinin receptors. Physiol Plant 154: 369–380 25263537 10.1111/ppl.12290

[kiaa034-B61] Kurakawa T , Ueda N, Maekawa M, Kobayashi K, Kojima M, Nagato Y, Sakakibara H, Kyozuka J (2007) Direct control of shoot meristem activity by a cytokinin-activating enzyme. Nature 445: 652–655 17287810 10.1038/nature05504

[kiaa034-B62] Kuroha T , Tokunaga H, Kojima M, Ueda N, Ishida T, Nagawa S, Fukuda H, Sugimoto K, Sakakibara H (2009) Functional analyses of LONELY GUY cytokinin-activating enzymes reveal the importance of the direct activation pathway in Arabidopsis. Plant Cell 21: 3152–3169 19837870 10.1105/tpc.109.068676 PMC2782294

[kiaa034-B63] Launay A , Cabassa-Hourton C, Eubel H, Maldiney R, Guivarc’h A, Crilat E, Planchais S, Lacoste J, Bordenave-Jacquemin M, Clément G et al (2019) Proline oxidation fuels mitochondrial respiration during dark-induced leaf senescence in Arabidopsis thaliana. J Exp Bot 70: 6203–6214 31504781 10.1093/jxb/erz351 PMC6859731

[kiaa034-B64] Liang XW , Zhang L, Natarajan SK, Becker DF (2013) Proline mechanisms of stress survival. Antioxid Redox Signal 19: 998–1011 23581681 10.1089/ars.2012.5074 PMC3763223

[kiaa034-B65] Liebsch D , Keech O (2016) Dark-induced leaf senescence: new insights into a complex light-dependent regulatory pathway. New Phytol 212: 563–570 27716940 10.1111/nph.14217

[kiaa034-B66] Lim PO , Kim HJ, Nam HG (2007) Leaf senescence. Annu Rev Plant Biol 58: 115–136 17177638 10.1146/annurev.arplant.57.032905.105316

[kiaa034-B67] Lim PO , Woo HR, Nam HG (2003) Molecular genetics of leaf senescence in Arabidopsis. Trends Plant Sci 8: 272–278 12818661 10.1016/S1360-1385(03)00103-1

[kiaa034-B68] Lin YJ , Cao ML, Xu CG, Chen H, Wei J, Zhang QF (2002) Cultivating rice with delaying led-senescence by P-SAG12-IPT gene transfonination. Acta Botanica Sinica 44: 1333–1338

[kiaa034-B69] Lisec J , Schauer N, Kopka J, Willmitzer L, Fernie AR (2006) Gas chromatography mass spectrometry-based metabolite profiling in plants. Nat Protoc 1: 387–396 17406261 10.1038/nprot.2006.59

[kiaa034-B70] Liu YD , Yin ZJ, Yu JW, Li J,, Wei HL, Han XL, Shen FF (2012) Improved salt tolerance and delayed leaf senescence in transgenic cotton expressing the Agrobacterium IPT gene. Biol Plant 56: 237–246

[kiaa034-B71] Love MI , Huber W, Anders S (2014) Moderated estimation of fold change and dispersion for RNA-seq data with DESeq2. Genome Biol 15: 550 25516281 10.1186/s13059-014-0550-8 PMC4302049

[kiaa034-B72] Luedemann A , von Malotky L, Erban A, Kopka J (2012) TagFinder: preprocessing software for the fingerprinting and the profiling of gas chromatography-mass spectrometry based metabolome analyses. Methods Mol Biol 860: 255–286 22351182 10.1007/978-1-61779-594-7_16

[kiaa034-B73] Ma M , Wang Q, Li Z, Cheng H, Li Z, Liu X, Song W, Appels R, Zhao H (2015) Expression of TaCYP78A3, a gene encoding cytochrome P450 CYP78A3 protein in wheat (*Triticum aestivum* L.), affects seed size. Plant J 83: 312–325 26043144 10.1111/tpj.12896

[kiaa034-B74] Ma QH , Liu YC (2009) Expression of isopentenyl transferase gene (ipt) in leaf and stem delayed leaf senescence without affecting root growth. Plant Cell Rep 28: 1759–1765 19820948 10.1007/s00299-009-0776-1

[kiaa034-B75] Mao C , Lu S, Lv B, Zhang B, Shen J, He J, Luo L, Xi D, Chen X, Ming F (2017) A rice NAC transcription factor promotes leaf senescence via ABA biosynthesis. Plant Physiol 174: 1747–1763 28500268 10.1104/pp.17.00542 PMC5490923

[kiaa034-B76] McCabe MS , Garratt LC, Schepers F, Jordi WJ, Stoopen GM, Davelaar E, van Rhijn JH, Power JB, Davey MR (2001) Effects of P(SAG12)-IPT gene expression on development and senescence in transgenic lettuce. Plant Physiol 127: 505–516 11598225 PMC125086

[kiaa034-B77] Medeiros DB , Barros KA, Barros JAS, Omena-Garcia RP, Arrivault S, Sanglard L, Detmann KC, Silva WB, Daloso DM, DaMatta FM et al (2017) Impaired malate and fumarate accumulation due to the mutation of the tonoplast dicarboxylate transporter has little effects on stomatal behavior. Plant Physiol 175: 1068–1081 28899959 10.1104/pp.17.00971 PMC5664473

[kiaa034-B78] Miyashita Y , Good AG (2008) NAD(H)-dependent glutamate dehydrogenase is essential for the survival of *Arabidopsis thaliana* during dark-induced carbon starvation. J Exp Bot 59: 667–680 18296429 10.1093/jxb/erm340

[kiaa034-B79] Miyoshi K , Ahn BO, Kawakatsu T, Ito Y, Itoh J, Nagato Y, Kurata N (2004) PLASTOCHRON1, a timekeeper of leaf initiation in rice, encodes cytochrome P450. Proc Natl Acad Sci U S A 101: 875–880 14711998 10.1073/pnas.2636936100 PMC321774

[kiaa034-B80] Morris K , MacKerness SA, Page T, John CF, Murphy AM, Carr JP, Buchanan-Wollaston V (2000) Salicylic acid has a role in regulating gene expression during leaf senescence. Plant J 23: 677–685 10972893 10.1046/j.1365-313x.2000.00836.x

[kiaa034-B81] Müller D , Leyser O (2011) Auxin, cytokinin and the control of shoot branching. Ann Bot 107: 1203–1212 21504914 10.1093/aob/mcr069 PMC3091808

[kiaa034-B82] Nam HG (1997) The molecular genetic analysis of leaf senescence. Curr Opin Biotechnol 8: 200–207 9079732 10.1016/s0958-1669(97)80103-6

[kiaa034-B83] Nguyen KH , Jordi W, Van Dun K, Schepers F, Davelaar E, Stoopen G, Dix PJ, Kane EJ (2008) Delayed senescence in cauliflower transformed with an autoregulated isopentenyl transferase gene. Int J Plant Sci 169: 339–347

[kiaa034-B84] Nishiyama R , Watanabe Y, Fujita Y, Le DT, Kojima M, Werner T, Vankova R, Yamaguchi-Shinozaki K, Shinozaki K, Kakimoto T et al (2011) Analysis of cytokinin mutants and regulation of cytokinin metabolic genes reveals important regulatory roles of cytokinins in drought, salt and abscisic acid responses, and abscisic acid biosynthesis. Plant Cell 23: 2169–2183 21719693 10.1105/tpc.111.087395 PMC3160038

[kiaa034-B85] Nishiyama R , Watanabe Y, Leyva-Gonzalez MA, Van Ha C, Fujita Y, Tanaka M, Seki M, Yamaguchi-Shinozaki K, Shinozaki K, Herrera-Estrella L et al (2013) Arabidopsis AHP2, AHP3, and AHP5 histidine phosphotransfer proteins function as redundant negative regulators of drought stress response. Proc Natl Acad Sci U S A 110: 4840–4845 23487796 10.1073/pnas.1302265110 PMC3606972

[kiaa034-B86] Pei ZM , Ghassemian M, Kwak CM, McCourt P, Schroeder JI (1998) Role of farnesyltransferase in ABA regulation of guard cell anion channels and plant water loss. Science 282: 287–290 9765153 10.1126/science.282.5387.287

[kiaa034-B87] Rashotte AM , Mason MG, Hutchison CE, Ferreira FJ, Schaller GE, Kieber JJ (2006) A subset of Arabidopsis AP2 transcription factors mediates cytokinin responses in concert with a two-component pathway. Proc Natl Acad Sci U S A 103: 11081–11085 16832061 10.1073/pnas.0602038103 PMC1544176

[kiaa034-B88] Richardson AD , Duigan SP, Berlyn GP (2002) An evaluation of noninvasive methods to estimate foliar chlorophyll content. New Phytol 153: 185–194

[kiaa034-B90] Rivero RM , Kojima M, Gepstein A, Sakakibara H, Mittler R, Gepstein S, Blumwald E (2007) Delayed leaf senescence induces extreme drought tolerance in a flowering plant. Proc Natl Acad Sci U S A 104: 19631–19636 18048328 10.1073/pnas.0709453104 PMC2148340

[kiaa034-B91] Sakakibara H (2006) Cytokinins: activity, biosynthesis, and translocation. Annu Rev Plant Biol 57: 431–449 16669769 10.1146/annurev.arplant.57.032905.105231

[kiaa034-B92] Salleh FM , Mariotti L, Spadafora ND, Price AM, Picciarelli P, Wagstaff C, Lombardi L, Rogers H (2016) Interaction of plant growth regulators and reactive oxygen species to regulate petal senescence in wallflowers (*Erysimum linifolium*). BMC Plant Biol 16: 77 27039085 10.1186/s12870-016-0766-8 PMC4818919

[kiaa034-B93] Sharma S , Verslues PE (2010) Mechanisms independent of abscisic acid (ABA) or proline feedback have a predominant role in transcriptional regulation of proline metabolism during low water potential and stress recovery. Plant Cell Environ 33: 1838–1851 20545884 10.1111/j.1365-3040.2010.02188.x

[kiaa034-B94] Su YH , Liu YB, Zhang XS (2011) Auxin–cytokinin interaction regulates meristem development. Mol Plant 4: 616–625 21357646 10.1093/mp/ssr007 PMC3146736

[kiaa034-B95] Sykorová B , Kuresová G, Daskalova S, Trcková M, Hoyerová K, Raimanová I, Motyka V, Trávnícková A, Elliott MC, Kamínek M (2008) Senescence-induced ectopic expression of the *A. tumefaciens* ipt gene in wheat delays leaf senescence, increases cytokinin content, nitrate influx, and nitrate reductase activity, but does not affect grain yield. J Exp Bot 59: 377–387 18267946 10.1093/jxb/erm319

[kiaa034-B96] Szabados L , Savouré A (2010) Proline: a multifunctional amino acid. Trends Plants Sci 15: 89–97 10.1016/j.tplants.2009.11.009 20036181

[kiaa034-B97] Takei K , Sakakibara H, Sugiyama T (2001) Identification of genes encoding adenylate isopentenyltransferase, a cytokinin biosynthesis enzyme, in *Arabidopsis thaliana*. J Biol Chem 276: 26405–26410 11313355 10.1074/jbc.M102130200

[kiaa034-B98] Takei K , Yamaya T, Sakakibara H (2004) Arabidopsis CYP735A1 and CYP735A2 encode cytokinin hydroxylases that catalyze the biosynthesis of trans-Zeatin. J Biol Chem 279: 41866–41872 15280363 10.1074/jbc.M406337200

[kiaa034-B99] Tanaka Y , Sano T, Tamaoki M, Nakajima N, Kondo N, Hasezawa S (2006) Cytokinin and auxin inhibit abscisic acid-induced stomatal closure by enhancing ethylene production in Arabidopsis. J Exp Bot 57: 2259–2266 16798847 10.1093/jxb/erj193

[kiaa034-B100] Tschoep H , Gibon Y, Carillo P, Armengaud P, Szecowka M, Nunes-Nesi A, Fernie AR, Koehl K, Stitt M (2009) Adjustment of growth and central metabolism to a mild but sustained nitrogen-limitation in Arabidopsis. Plant Cell Environ 32: 300–318 19054347 10.1111/j.1365-3040.2008.01921.x

[kiaa034-B101] Ueda H , Kusaba M (2015) Strigolactone regulates leaf senescence in concert with ethylene in Arabidopsis. Plant Physiol 169: 138–147 25979917 10.1104/pp.15.00325 PMC4577378

[kiaa034-B102] Vogelmann K , Drechsel G, Bergler J, Subert C, Philippar K, Soll J, Engelmann JC, Engelsdorf T, Voll LM, Hoth S (2012) Early senescence and cell death in Arabidopsis saul1 mutants involves the PAD4-dependent salicylic acid pathway. Plant Physiol 159: 1477–1487 22706448 10.1104/pp.112.196220 PMC3425192

[kiaa034-B103] Wang JW , Schwab R, Czech B, Mica E, Weigel D (2008) Dual effects of miR156-targeted SPL genes and CYP78A5/KLUH on plastochron length and organ size in *Arabidopsis thaliana*. Plant Cell 20: 1231–1243 18492871 10.1105/tpc.108.058180 PMC2438454

[kiaa034-B104] Watanabe M , Balazadeh S, Tohge T, Erban A, Giavalisco P, Kopka J, Mueller-Roeber B, Fernie AR, Hoefgen R (2013) Comprehensive dissection of spatiotemporal metabolic shifts in primary, secondary, and lipid metabolism during developmental senescence in Arabidopsis. Plant Physiol 162: 1290–1310 23696093 10.1104/pp.113.217380 PMC3707545

[kiaa034-B105] Weaver LM , Gan SS, Quirino B, Amasino RM (1998) A comparison of the expression patterns of several senescence-associated genes in response to stress and hormone treatment. Plant Mol Biol 37: 455–469 9617813 10.1023/a:1005934428906

[kiaa034-B107] Werner T , Motyka V, Laucou V, Smets R, Van Onckelen H, Schmülling T (2003) Cytokinin-deficient transgenic Arabidopsis plants show multiple developmental alterations indicating opposite functions of cytokinins in the regulation of shoot and root meristem activity. Plant Cell 15: 2532–2550 14555694 10.1105/tpc.014928 PMC280559

[kiaa034-B108] Wingler A (2011) Interactions between flowering and senescence regulation and the influence of low temperature in Arabidopsis and crop plants. Ann Appl Biol 159: 320–338

[kiaa034-B110] Wohlbach DJ , Quirino BF, Sussman MR (2008) Analysis of the Arabidopsis histidine kinase ATHK1 reveals a connection between vegetative osmotic stress sensing and seed maturation. Plant Cell 20: 1101–1117 18441212 10.1105/tpc.107.055871 PMC2390728

[kiaa034-B111] Woo HR , Chung KM, Park JH, Oh SA, Ahn T, Hong SH, Jang SK, Nam HG (2001) ORE9, an F-box protein that regulates leaf senescence in Arabidopsis. Plant Cell 13: 1779–1790 11487692 10.1105/TPC.010061 PMC139127

[kiaa034-B112] Woo HR , Kim HJ, Nam HG, Lim PO (2013) Plant leaf senescence and death—regulation by multiple layers of control and implications for aging in general. J Cell Sci 126: 4823–4833 24144694 10.1242/jcs.109116

[kiaa034-B114] Yang WB , Gao MJ,, Yin X, Liu JY, Xu YH, Zeng LJ, Li Q, Zhang SB, Wang JM, Zhang XM et al (2013) Control of rice embryo development, shoot apical meristem maintenance, and grain yield by a novel cytochrome P450. Mol Plant 6: 1945–1960 23775595 10.1093/mp/sst107

[kiaa034-B115] Yolcu S , Li X, Li S, Kim YJ (2018) Beyond the genetic code in leaf senescence. J Exp Bot 69: 801–810 29253191 10.1093/jxb/erx401

[kiaa034-B116] Yoshida S (2003) Molecular regulation of leaf senescence. Curr Opin Plant Biol 6: 79–84 12495755 10.1016/s1369526602000092

[kiaa034-B117] Yoshimoto K , Jikumaru Y, Kamiya Y, Kusano M, Consonni C, Panstruga R, Ohsumi Y, Shirasu K (2009) Autophagy negatively regulates cell death by controlling NPR1-dependent salicylic acid signaling during senescence and the innate immune response in Arabidopsis. Plant Cell 21: 2914–2927 19773385 10.1105/tpc.109.068635 PMC2768913

[kiaa034-B118] Yu G , Wang LG, Han Y, He QY (2012) clusterProfiler: an R package for comparing biological themes among gene clusters. OMICS 16: 284–287 22455463 10.1089/omi.2011.0118 PMC3339379

[kiaa034-B119] Zhang K , Halitschke R, Yin C, Liu CJ, Gan SS (2013) Salicylic acid 3-hydroxylase regulates Arabidopsis leaf longevity by mediating salicylic acid catabolism. Proc Natl Acad Sci U S A 110: 14807–14812 23959884 10.1073/pnas.1302702110 PMC3767541

[kiaa034-B120] Zhang S , Li C, Wang R, Chen Y, Shu S, Huang R, Zhang D, Li J, Xiao S, Yao N et al (2017) The Arabidopsis mitochondrial protease FtSH4 is involved in leaf senescence via regulation of WRKY-dependent salicylic acid accumulation and signaling. Plant Physiol 173: 2294–2307 28250067 10.1104/pp.16.00008 PMC5373041

[kiaa034-B121] Zhao LH , Cai HY, Su ZX, Wang LL,, Huang XY, Zhang M, Chen PJ, Dai XZ, Zhao HM, Palanivelu R et al (2018) KLU suppresses megasporocyte cell fate through SWR1-mediated activation of WRKY28 expression in Arabidopsis. Proc Natl Acad Sci U S A 115: E526–E535 29288215 10.1073/pnas.1716054115 PMC5776990

[kiaa034-B122] Zhao XY , Wang JG, Song SJ, Wang Q, Kang H, Zhang Y, Li S (2016) Precocious leaf senescence by functional loss of PROTEIN S-ACYL TRANSFERASE14 involves the NPR1-dependent salicylic acid signaling. Sci Rep 6: 20309 26842807 10.1038/srep20309 PMC4740857

[kiaa034-B123] Zhao Y , Chan Z, Gao J, Xing L, Cao M, Yu C, Hu Y, You J, Shi H, Zhu Y et al (2016) ABA receptor PYL9 promotes drought resistance and leaf senescence. Proc Natl Acad Sci U S A 113: 1949–1954 26831097 10.1073/pnas.1522840113 PMC4763734

[kiaa034-B124] Zondlo SC , Irish VF (1999) CYP78A5 encodes a cytochrome P450 that marks the shoot apical meristem boundary in Arabidopsis. Plant J 19: 259–268 10476073 10.1046/j.1365-313x.1999.00523.x

[kiaa034-B125] Zwack PJ , Rashotte AM (2013) Cytokinin inhibition of leaf senescence. Plant Signal Behav 8: e24737 23656876 10.4161/psb.24737 PMC3908980

[kiaa034-B126] Zwack PJ , Robinson BR, Risley MG, Rashotte AM (2013) Cytokinin response factor 6 negatively regulates leaf senescence and is induced in response to cytokinin and numerous abiotic stresses. Plant Cell Physiol 54: 971–981 23539244 10.1093/pcp/pct049

