# Supplementary material for: Multi-omics approach reveals the contribution of KLU to leaf longevity and drought tolerance
Source: Plant Physiol. 2020 Nov 28;185(2):352–68. doi: 10.1093/plphys/kiaa034 (PMC8133585; doi:10.1093/plphys/kiaa034)
Supplement: kiaa034_Supplementary_Data [file kiaa034_supplementary_data.zip › pp.01345.2020-s01.pdf]

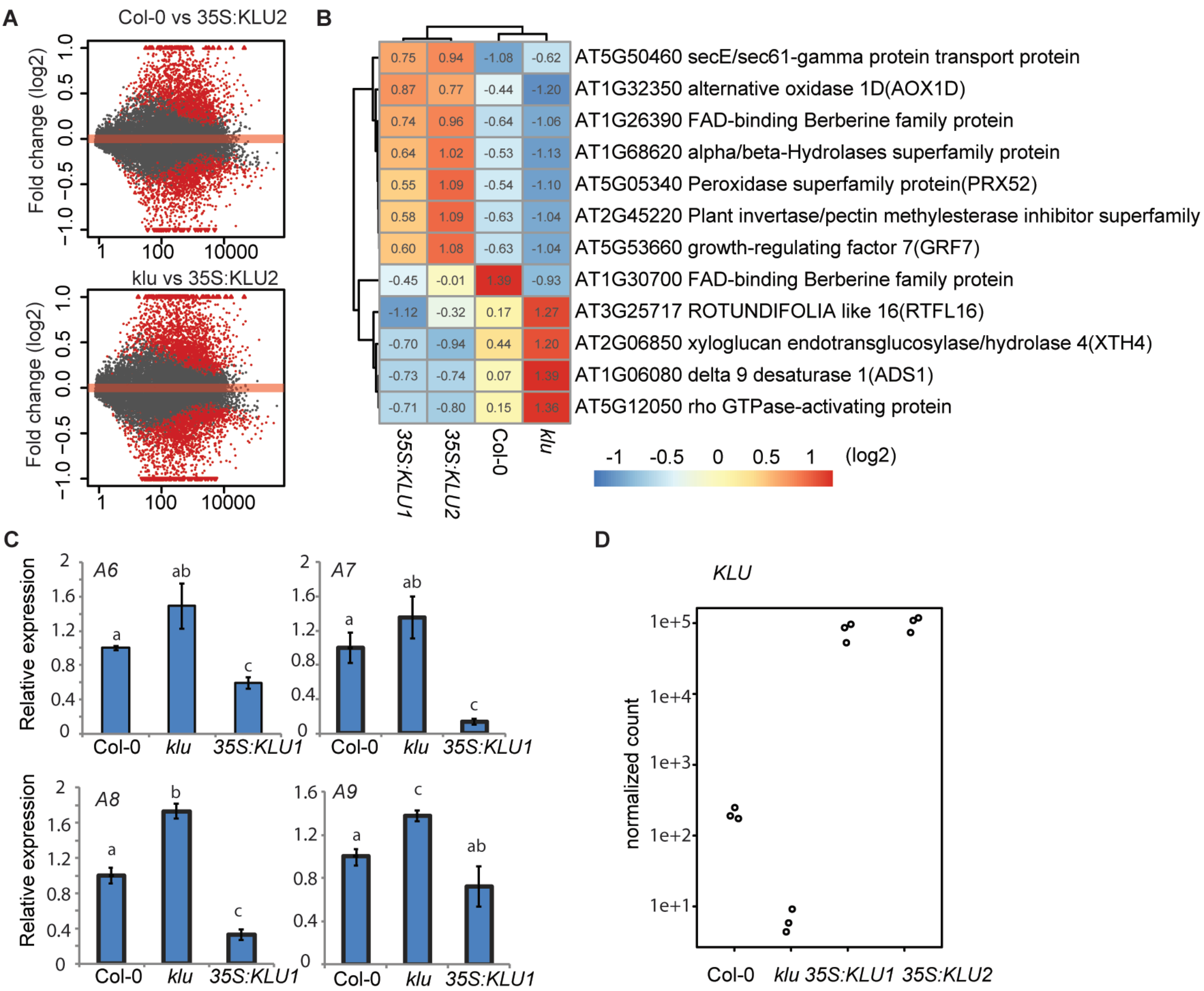

Supplemental Figure S1. Overview of the transcriptome changes and the expression of CYP78A5 family genes.

A, MA-plot show log2 fold change (y-axis) of Col-0 vs *35S:KLU2* comparison and *klu* vs *35S:KLU2* comparison over the mean of normalized counts (x-axis) for all the samples. Red points indicate adjusted *p* value is less than 0.05. Points falling out of the window are plotted as triangles pointing either up or down. B, A heatmap matrix based on the FPKM of the genes shared from DEGs of Col-0 vs OE, *klu* vs OE and Col-0 vs *klu*. C, The relative transcript abundance of other *CYP78A5* family members in the aerial part of Col-0, *klu* and *35S:KLU1* at 28 DAG. The *Arabidopsis UBIQUITIN 5* was used as an internal control. Error bars represent SD. D, The normalized counts of *KLU* in the Col-0, *klu*, *35S:KLU1* and *35S:KLU2*.

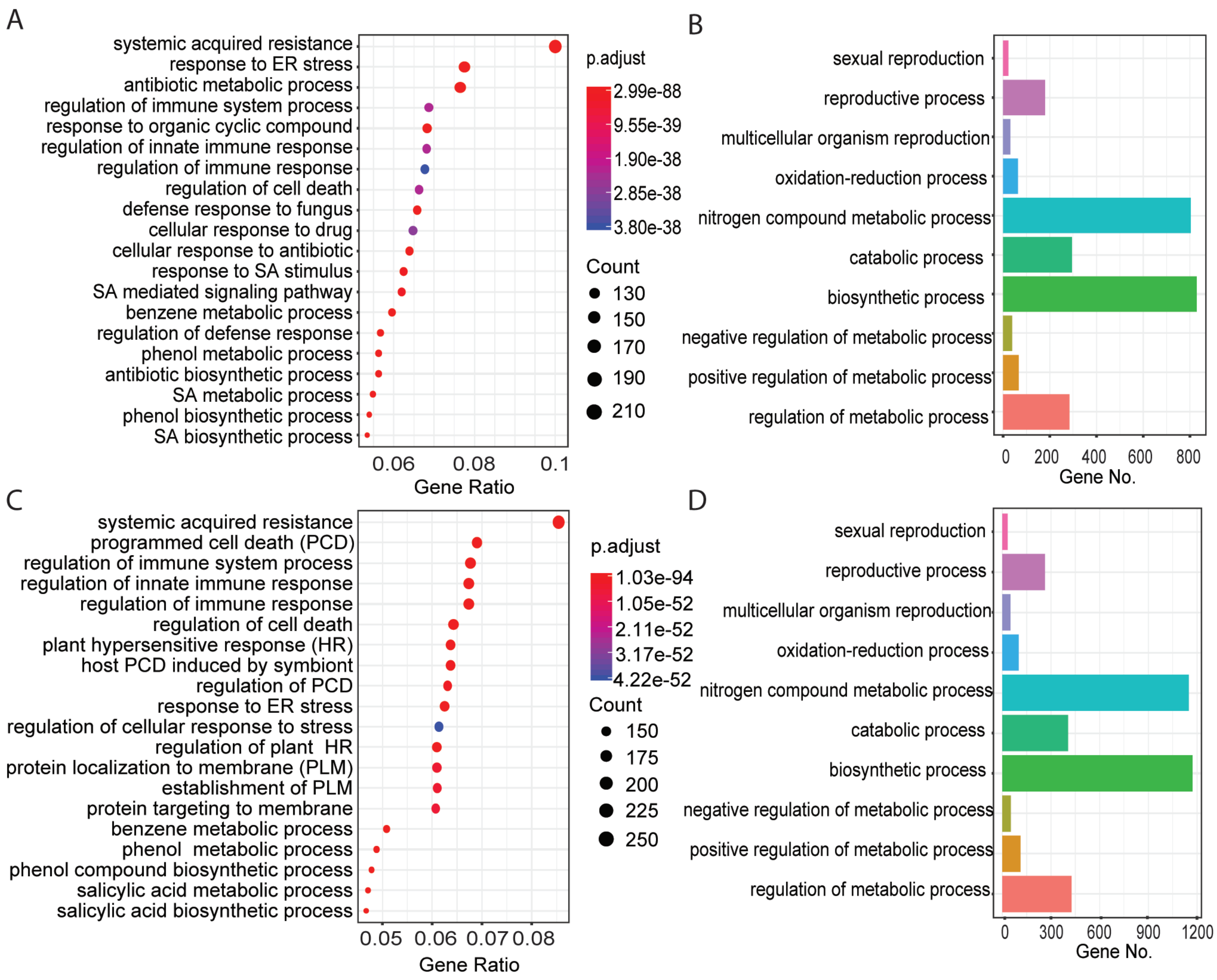

Supplemental Figure S2. GO enrichment analysis.

A, Top 20 enriched GO terms for DEGs from Col-0 vs OE (adjust *p*-vale < 0.01). B, Top10 GO categories at the third levels of the GO tress for DEGs of Col-0 vs OE (adjust *p*-vale < 0.01). C, Top 20 enriched GO terms for DEGs from *klu* vs OE (adjust *p*-vale < 0.01). B, Top 10 GO categories at the third levels of the GO tress for DEGs of *klu* vs OE (adjust *p*-vale < 0.01).

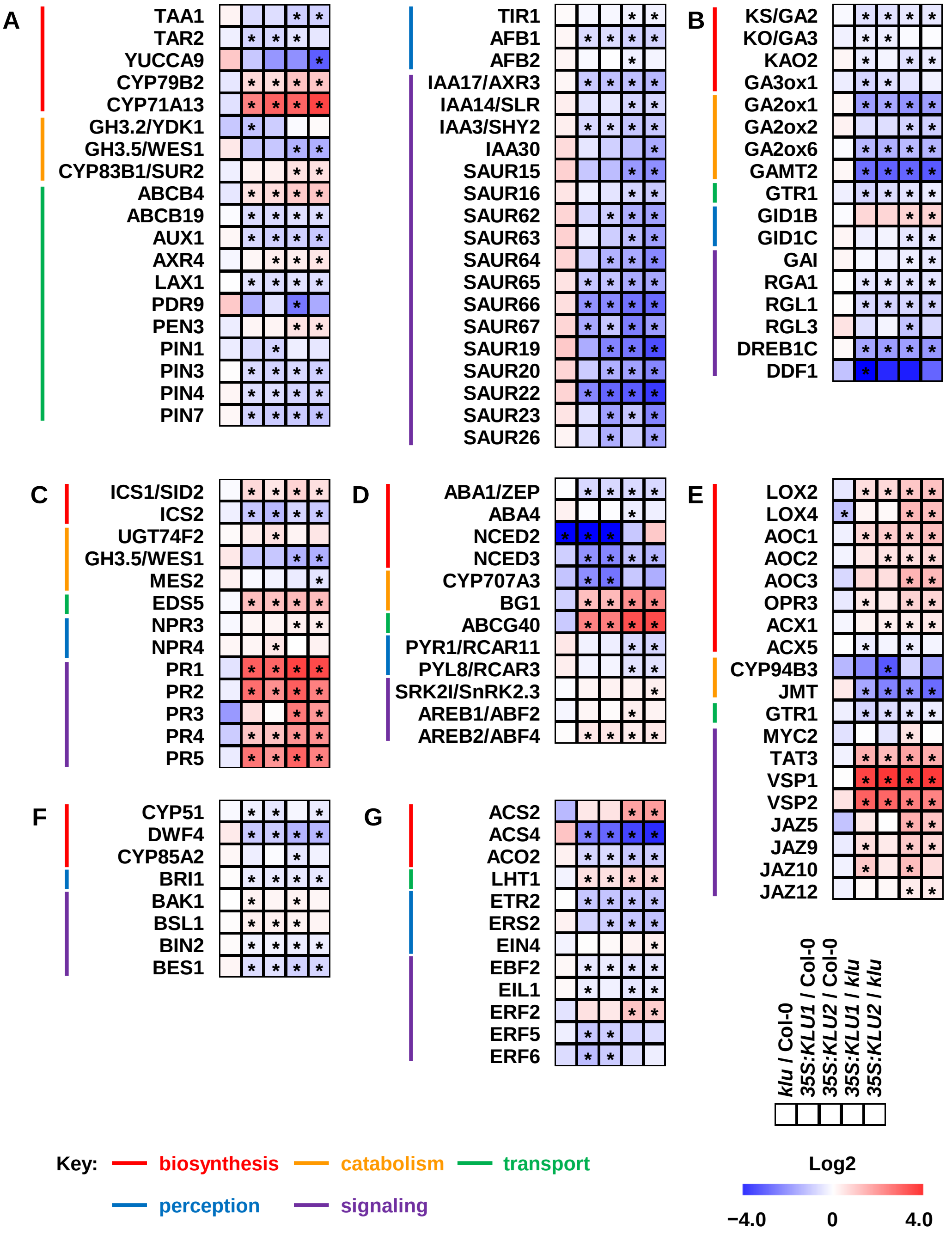

Supplemental Figure S3. The expression of the genes associated with plant hormone metabolism, transport, perception, and signaling in *klu* and *35S:KLU* plants anlyzed by RNA-sequencing. (A) auxin, (B) GA, (C) SA, (D) ABA, (E) JA, (F) BR, and (G) ethylene. Asterisks indicate the differentially expressed genes (DEGs) (adjusted p-value < 0.05). The detailed expression data is available in Supplemental Tables S3-S9.

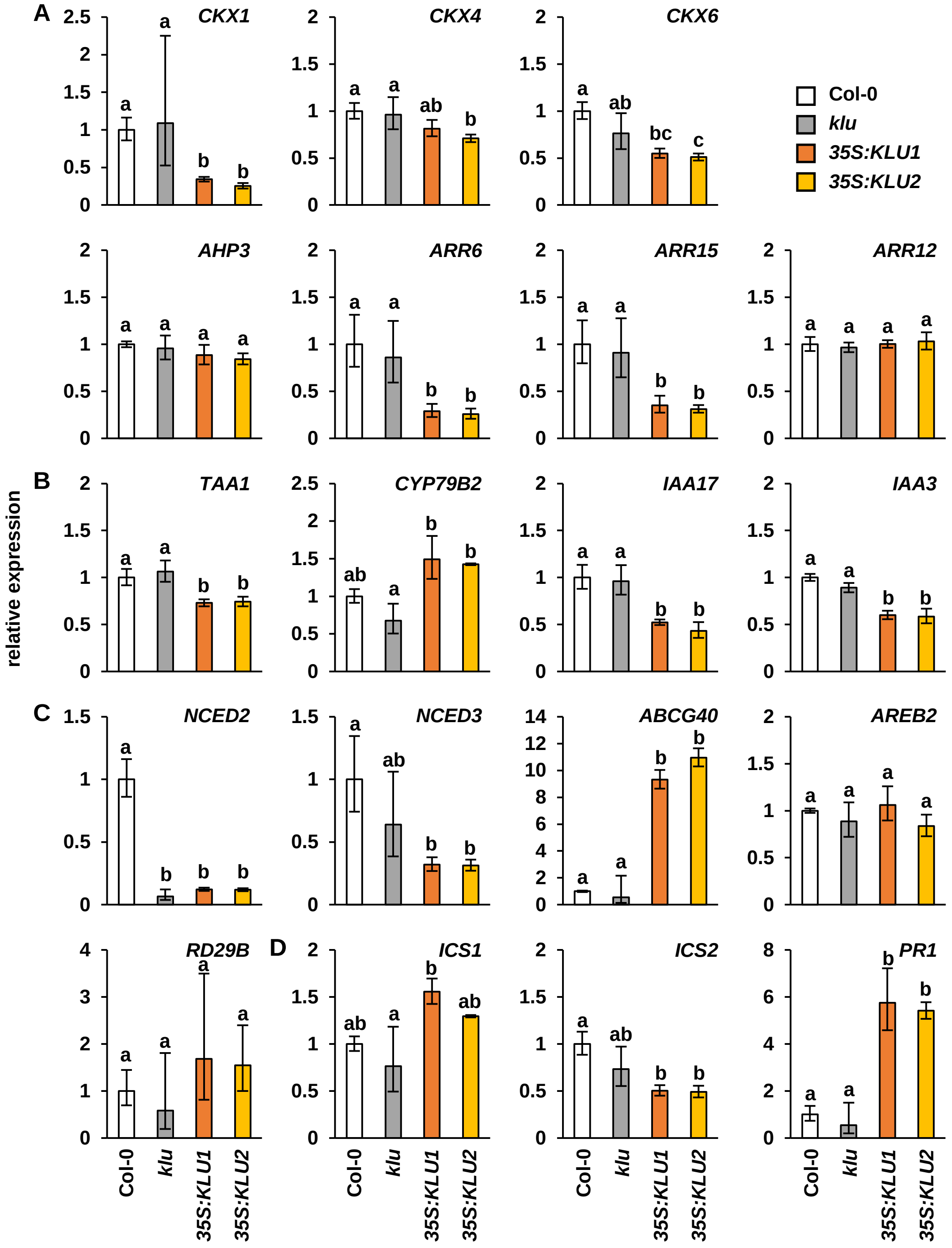

Supplemental Figure S4. The expression of the selected genes associated with plant hormone metabolism, transport, perception, and signaling in *klu* and *35S:KLU* plants analyzed by RT-qPCR. (A) cytokinin, (B) auxin, (C) ABA, and (D) SA. Relative expression levels were calculated by the comparative C_T method with *ACT2* as a reference gene. For each gene, the expression level in Col-0 was defined as 1. Data represent means and SD of three biological replicates (three or four replicate reactions for each). Different letters denote statistically significant differences (*P* < 0.05) by single-factor ANOVA followed by a Tukey-Kramer post-hoc test.

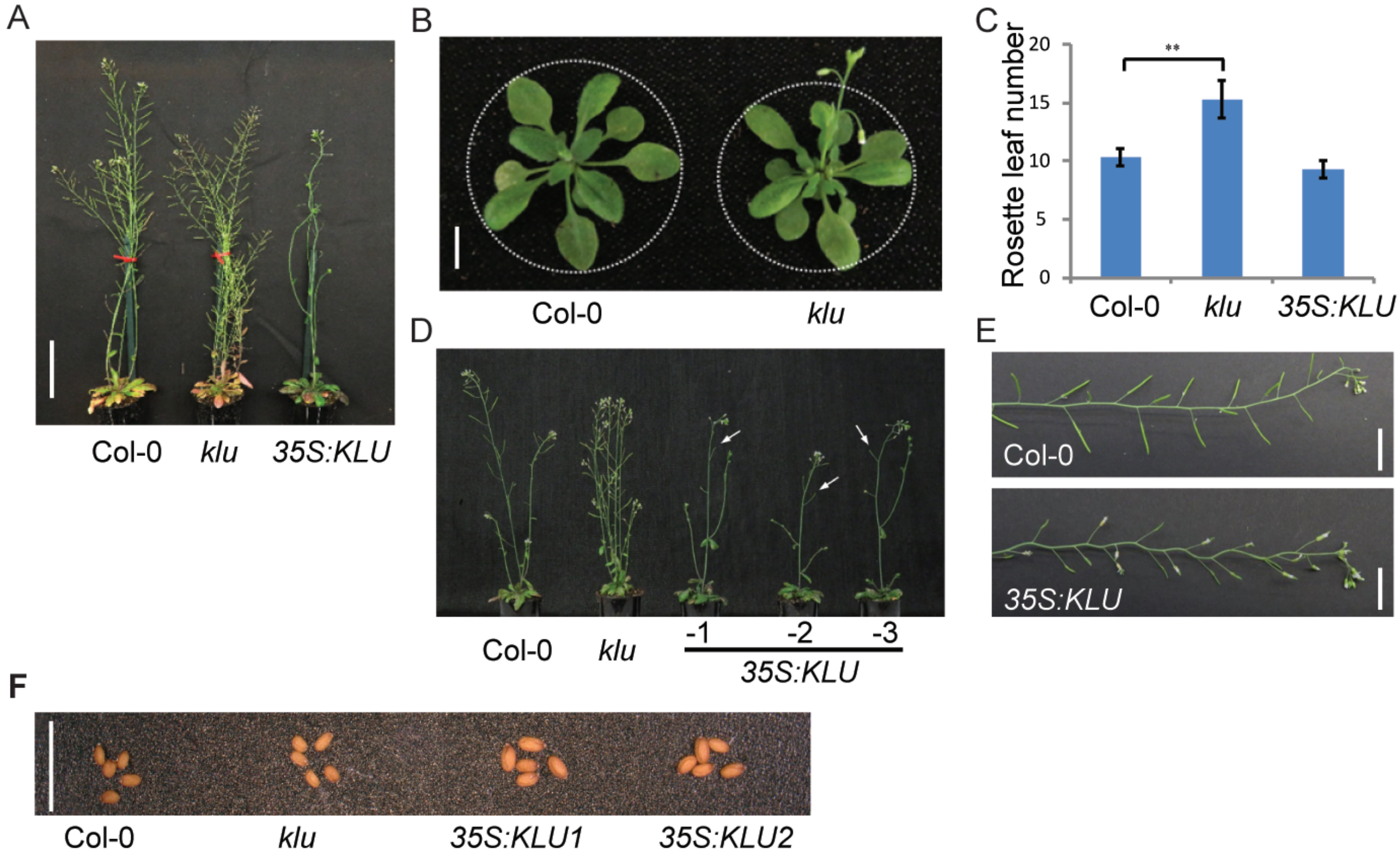

Supplemental Figure S5. Phenotypes of *klu* and *KLU*-overexpression plants.

A, The gross phenotype of wild-type (WT), *klu*, and *35S:KLU* plants at reproductive stag. Scale bar, 6 cm. B, *klu* show early flowering time. Scale bar, 1cm. C, Rosette leaf number in WT, *klu*, and *35S:KLU* plants at the 28 DAG in the short day condition. Error bars represent SD (n = 12). Student's t-test, $p < 0.01$. D, The representative phenotype of WT, *klu*, and three indivual *35S:KLU* plants at reproductive stage. Scale bar, 6 cm. E, The representative stem phenotype of WT and *35S:KLU* plants at reproductive stage. Scale bar, 1 cm. F, The seed phenotype of WT, *klu*, and *35S:KLU*. Scale bar, 0.2 cm.

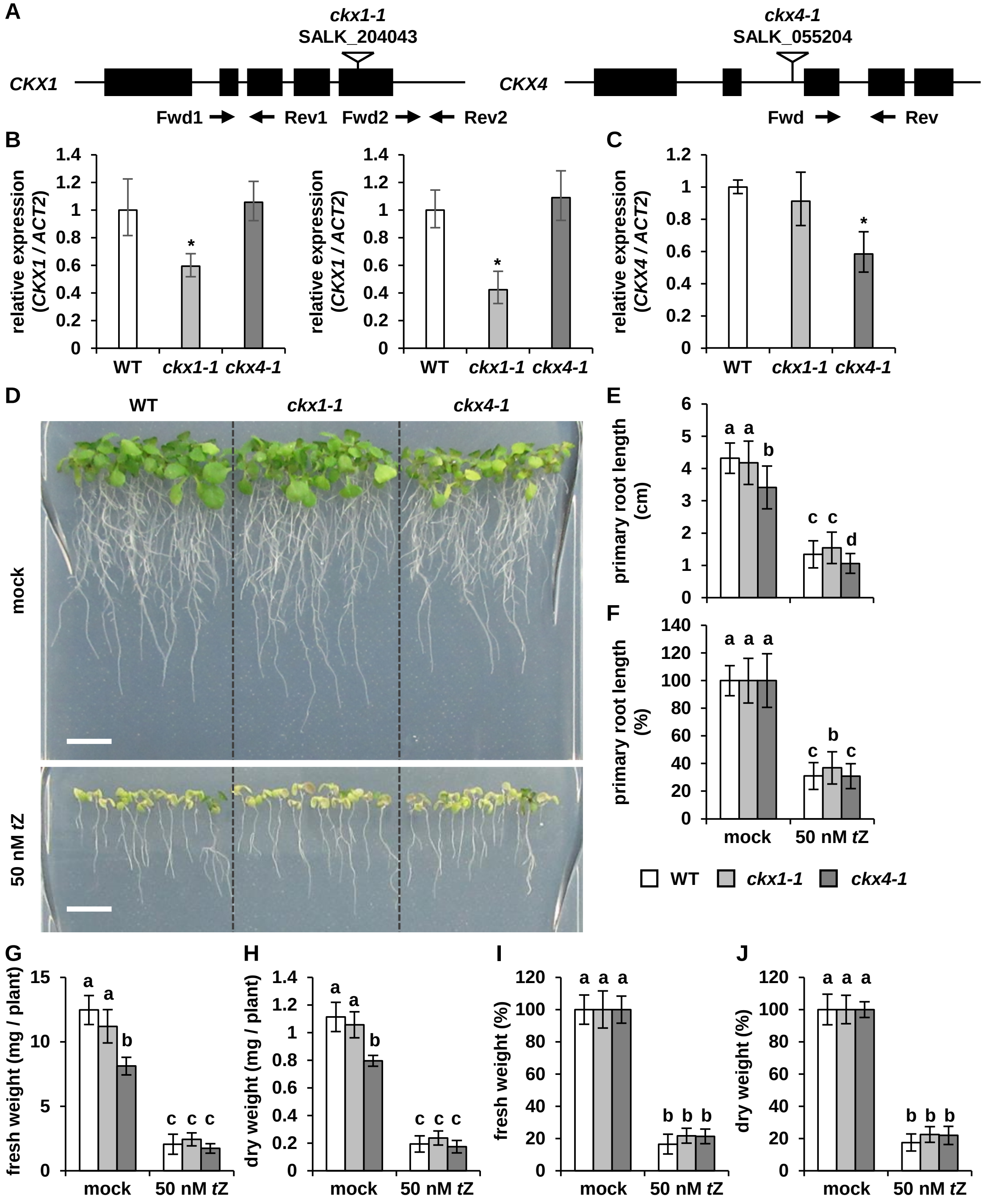

Supplemental Figure S6. Growth characteristics of the *ckx* mutants.

(A) Scheme of the *CKX* genes. Positions of the T-DNA insertions and the primers used in (B and C) are shown. Exons are indicated as filled boxes. (B, C) Expression of the *CKX*s analyzed by RT-qPCR. Total RNAs were extracted from 12-day-old seedlings grown on agar plates under long-day conditions. Relative expression levels were calculated by the comparative C_T method with *ACT2* as a reference gene. For each gene, the expression level in WT was defined as 1. For *CKX1*, two primer sets (1 and 2 in A) were used to verify the decreased expression in the T-DNA line (left and right panels, respectively). Data represent means and SD of four replicate reactions. * $P < 0.01$ (*t*-test, comparison with WT). (D to J) Growth of the *ckx* mutants on plates supplemented with cytokinin. Seeds (n = 12 for each) were sown on agar plates (0.5 × MS with 1% sucrose) with or without 50 nM *trans*-zeatin (*tZ*) and then stratified at 4ºC for 3 days. Plants were grown vertically under long-day conditions for two weeks. (D) The photographs show seedlings grown on agar plates without or with 50 nM *tZ*. White bars = 1 cm. (E, F) Primary root lengths of two-week-old seedlings. The graph shows the result of four biological replicates (WT mock, n = 46; WT *tZ*, n = 45; *ckx1* mock, n = 47; *ckx1 tZ*, n = 47; *ckx4* mock, n = 47; *ckx4 tZ*, n = 48). Error bars represent SD. Relative root lengths of plants grown on plates with cytokinin were shown as a percentage (F). (G to J) Fresh and dry weights of two-week-old seedlings. Seedlings ($n \geq 10$) were pooled to measure weights and then the total weights were divided by the number of plants. Data represent means and SD of four biological replicates. Relative weights of plants grown on plates with cytokinin were shown as a percentage (I, J). Different letters indicate statistically significant differences ($P < 0.05$) by one-way ANOVA followed by a Tukey's post-hoc test.

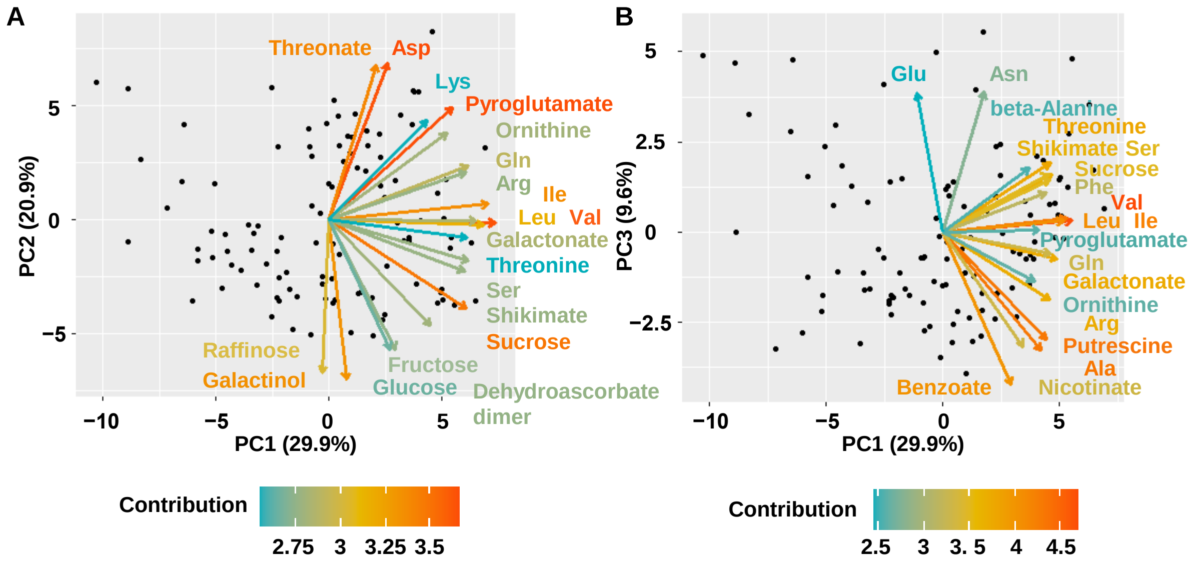

Supplementary Figure S7. Top metabolites contributing the PCA plots.

Top 20 metabolites contributing the PCA plots (Figure 5A,B) were shown by colored arrows. Black dots indicate individual plots as shown in Figure 5A,B.

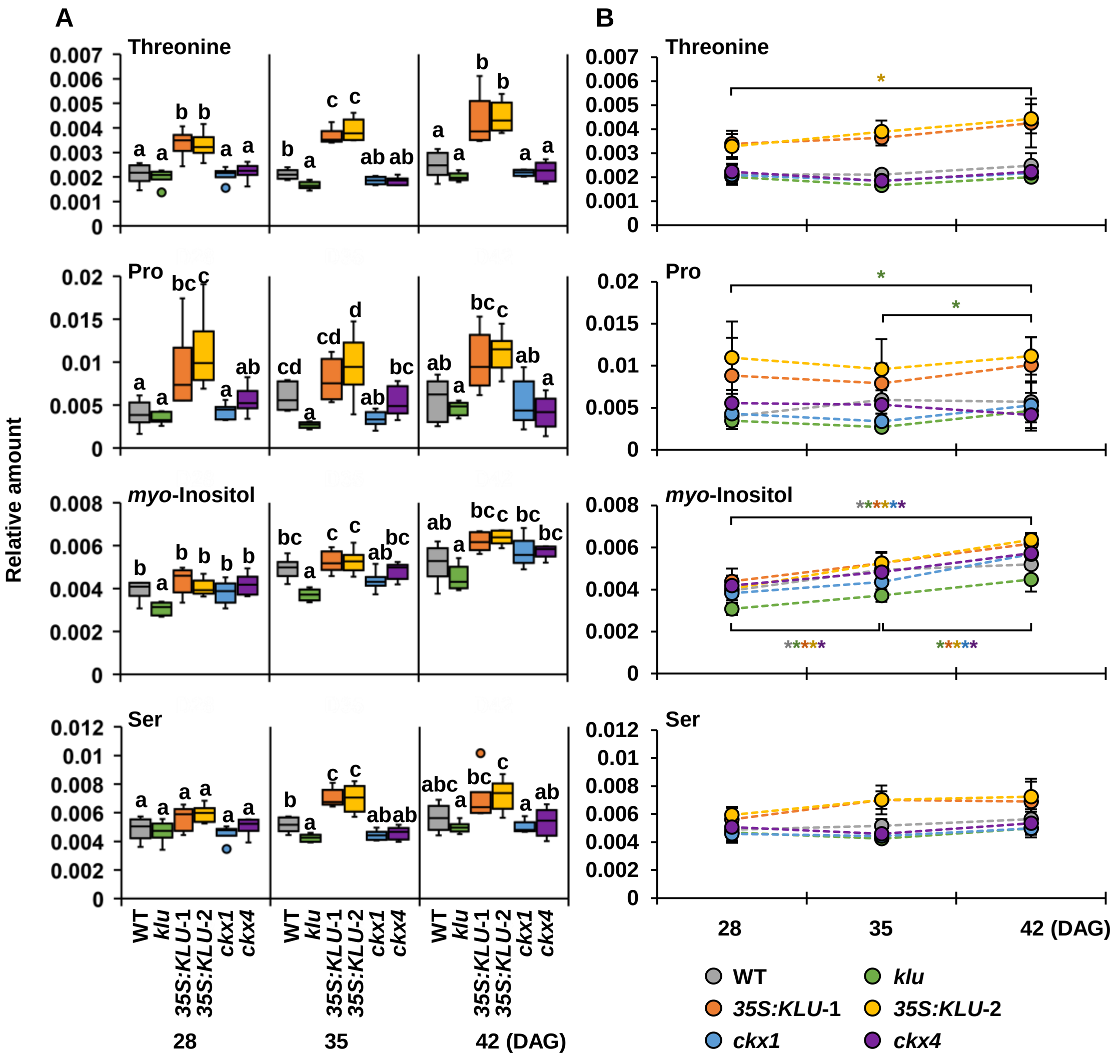

Supplementary Figure S8. The metabolites showed constitutive increased-accumulation in the *KLU*-overexpressing lines.

The primary metabolites that showed significant differences between *klu* mutant and the *KLU*-overexpressing lines at more than two time points are displayed. The relative amounts of primary metabolites determined by GC-MS are shown in box and whisker plots (A) and line charts (B). (A) Different letters denote statistically significant differences (*P* < 0.05) within each time point by one-way ANOVA followed by a Tukey's post-hoc test. (B) Bars indicate SD (*n* = 5 or 6). Colored asterisks indicate statistically significant differences (*P* < 0.05) in each line by one-way ANOVA followed by a Tukey's post-hoc test.

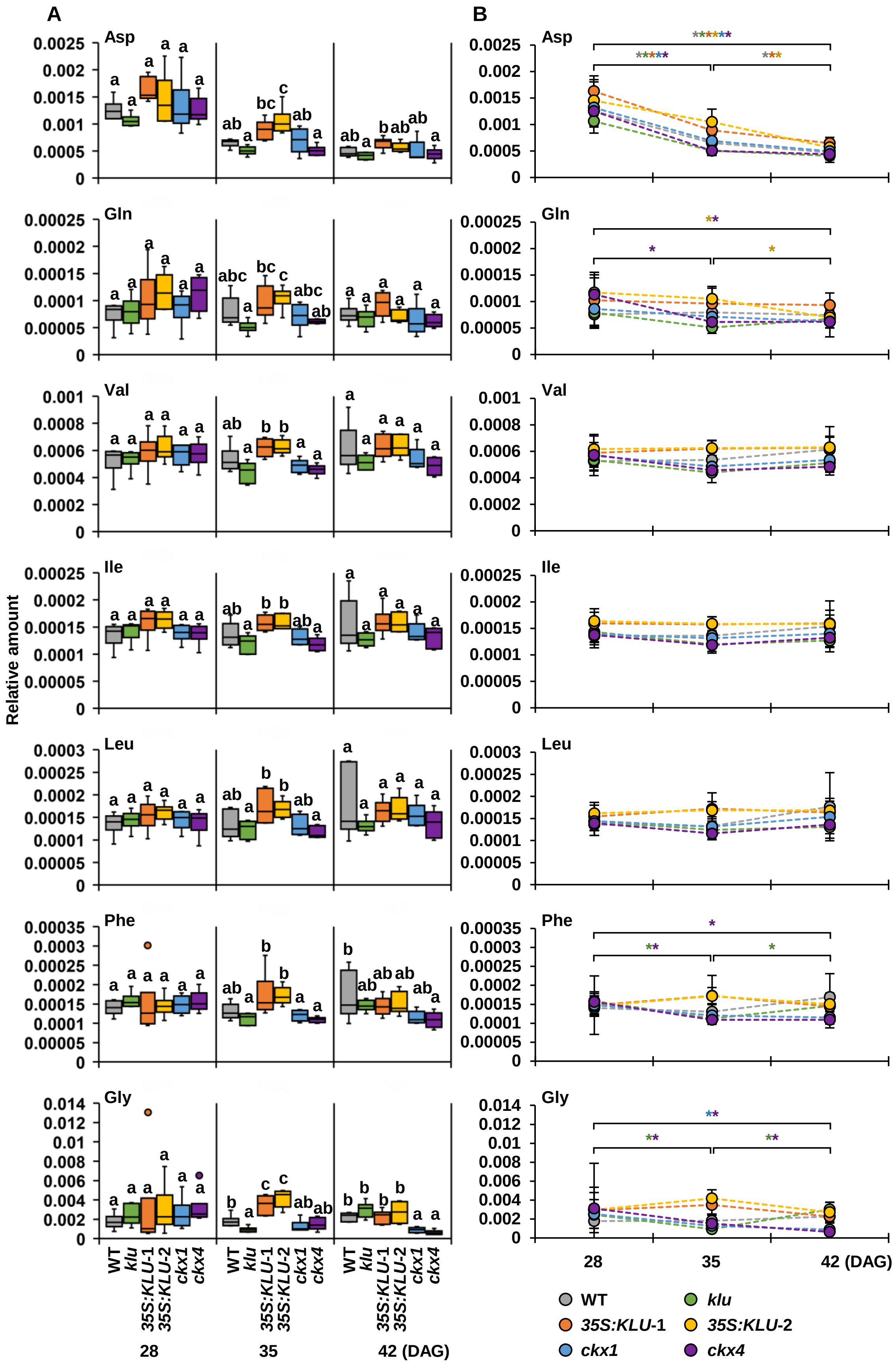

Supplementary Figure S9. The proteinogenic amino acids more accumulated in the *KLU*-overexpressing lines than in the *klu* mutant.

The relative amounts of primary metabolites determined by GC-MS are shown in box and whisker plots (A) and line charts (B). (A) Different letters denote statistically significant differences (P < 0.05) within each time point by one-way ANOVA followed by a Tukey's post-hoc test. (B) Bars indicate SD (n = 5 or 6). Colored asterisks indicate statistically significant differences (P < 0.05) in each line by one-way ANOVA followed by a Tukey's post-hoc test.

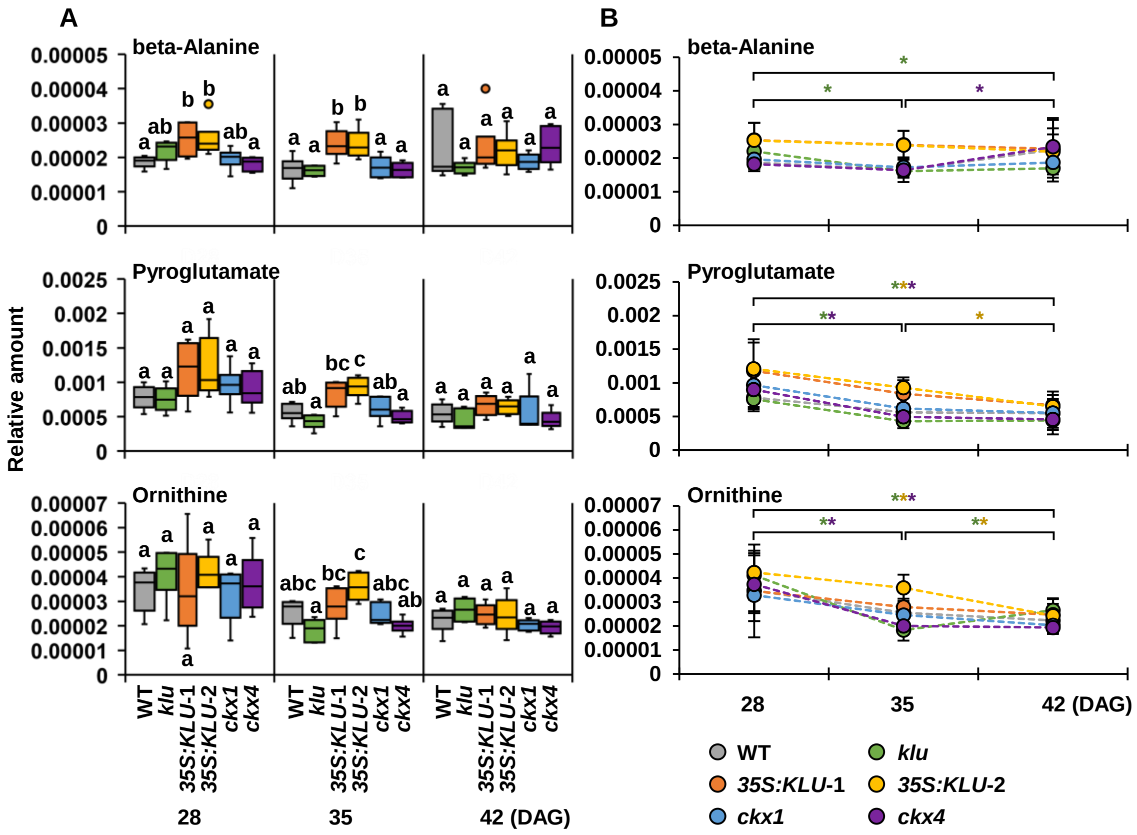

Supplementary Figure S10. The non-proteinogenic amino acids more accumulated in the *KLU*-overexpressing lines than in the *klu* mutant.
The relative amounts of primary metabolites determined by GC-MS are shown in box and whisker plots (A) and line charts (B). (A) Different letters denote statistically significant differences ($P < 0.05$) within each time point by one-way ANOVA followed by a Tukey's post-hoc test. (B) Bars indicate SD (n = 5 or 6). Colored asterisks indicate statistically significant differences ($P < 0.05$) in each line by one-way ANOVA followed by a Tukey's post-hoc test.

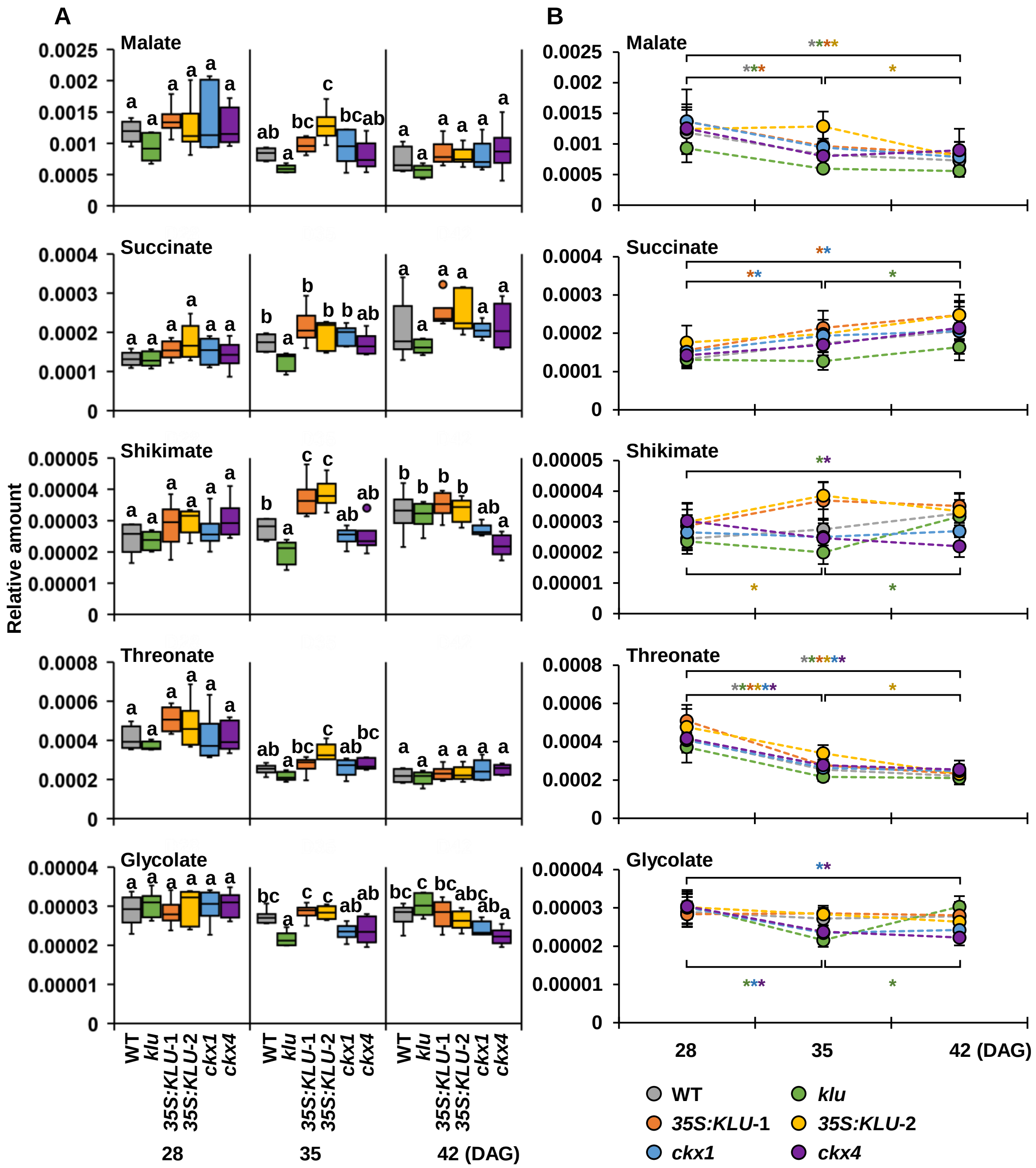

Supplementary Figure S11. The organic acids more accumulated in the *KLU*-overexpressing lines than in the *klu* mutant.

The relative amounts of primary metabolites determined by GC-MS are shown in box and whisker plots (A) and line charts (B). (A) Different letters denote statistically significant differences (*P* < 0.05) within each time point by one-way ANOVA followed by a Tukey's post-hoc test. (B) Bars indicate SD (*n* = 5 or 6). Colored asterisks indicate statistically significant differences (*P* < 0.05) in each line by one-way ANOVA followed by a Tukey's post-hoc test.

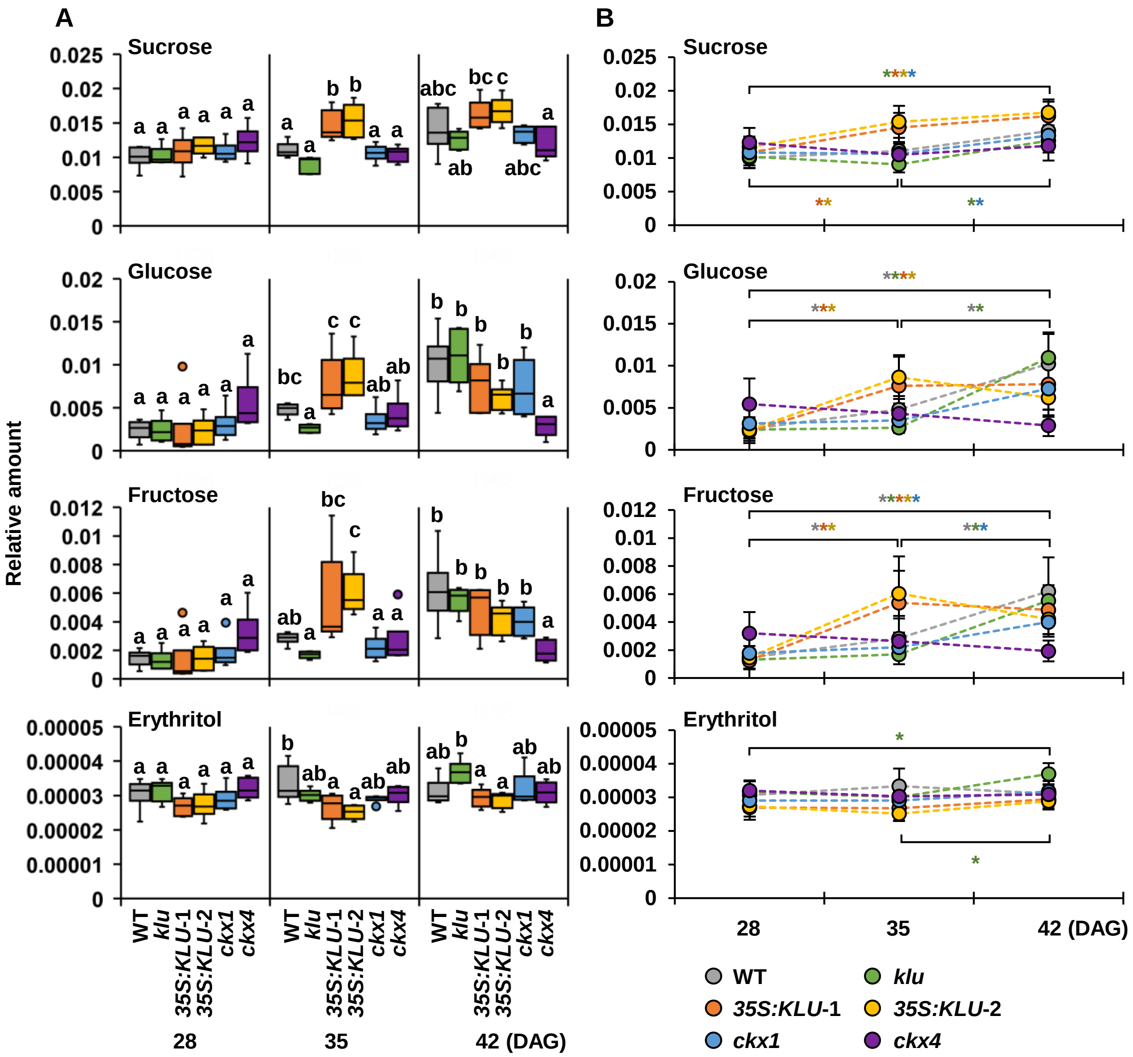

Supplementary Figure S12. The levels of sugars and sugar alcohols were altered in the *KLU*-overexpressing lines compared to in the *klu* mutant.

The relative amounts of primary metabolites determined by GC-MS are shown in box and whisker plots (A) and line charts (B). (A) Different letters denote statistically significant differences (*P* < 0.05) within each time point by one-way ANOVA followed by a Tukey's post-hoc test. (B) Bars indicate SD (*n* = 5 or 6). Colored asterisks indicate statistically significant differences (*P* < 0.05) in each line by one-way ANOVA followed by a Tukey's post-hoc test.

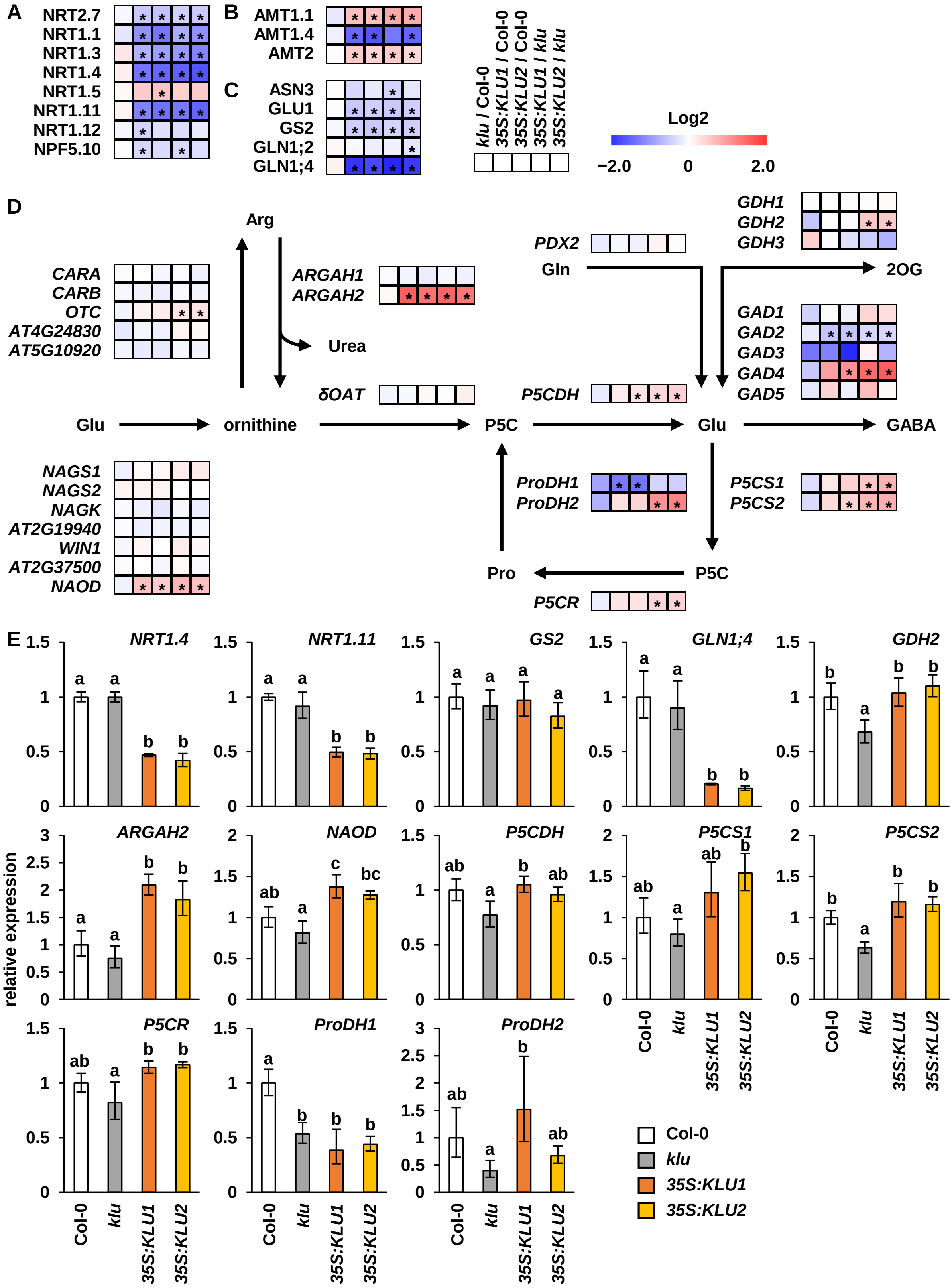

Supplemental Figure S13. The expression of the genes associated with nitrogen assimilation and proline metabolism in *klu* and *35S:KLU* plants analyzed by RNA-sequencing. (A) nitrate transport, (B) ammonium transport, (C) nitrogen assimilation and (D) proline metabolism. Asterisks indicate the differentially expressed genes (DEGs) (adjusted p-value < 0.05). The detailed expression data is available in Supplemental Tables S10 and S11. (E) The expression of the selected genes analyzed by RT-qPCR. Relative expression levels were calculated by the comparative C_T method with *ACT2* as a reference gene. For each gene, the expression level in Col-0 was defined as 1. Data represent means and SD of three biological replicates (three or four replicate reactions for each). Different letters denote statistically significant differences ($P < 0.05$) by single-factor ANOVA followed by a Tukey-Kramer post-hoc test. 2OG, 2-oxoglutarate; GABA, 4-aminobutyric acid; P5C, pyrroline-5-carboxylate.

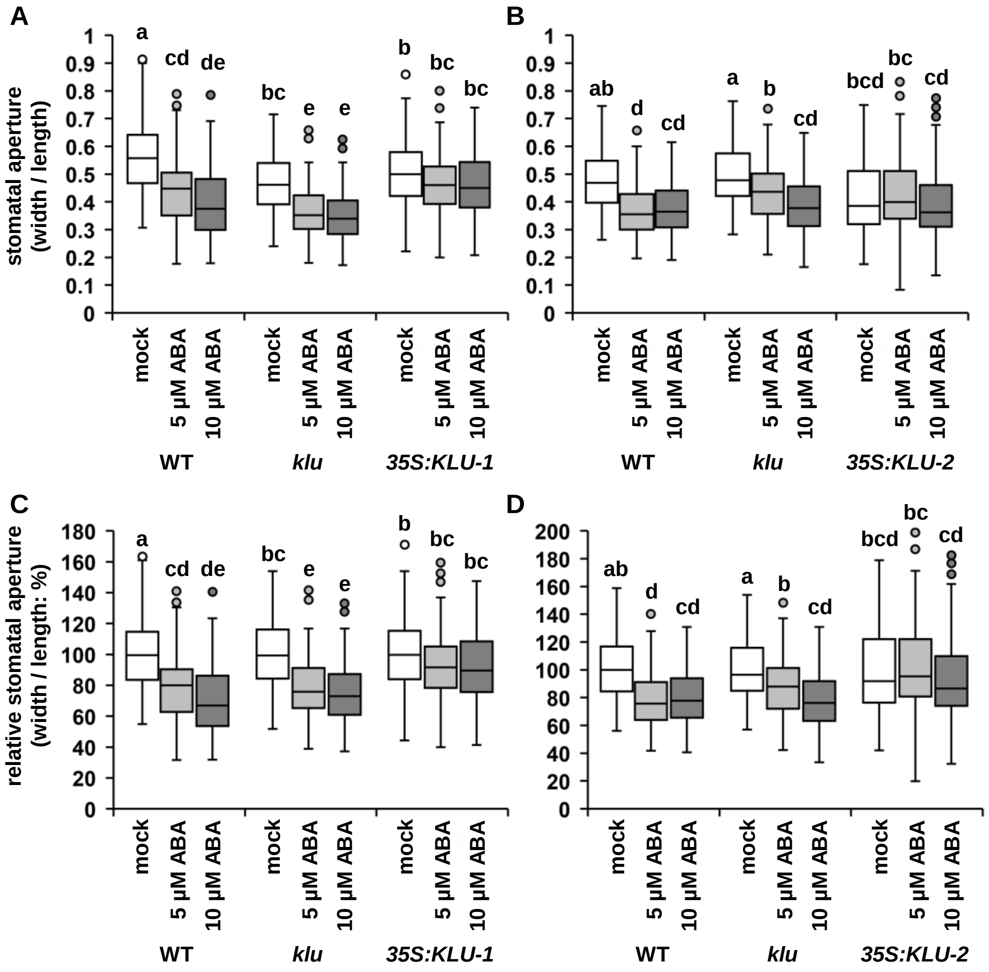

Supplemental Figure S14. Stomatal closure of guard cells in response to ABA.
Rosette leaves detached from 6-week-old plants were treated with or without ABA for 3 h after incubation in stomatal opening buffer for 2 h. Two independent experiments of different *35S:KLU* lines were performed. (A, B) The box and whisker plots represent data from four or five biological replicates (A, WT mock, *n* = 100; WT 5 μM ABA, *n* = 99; WT 10 μM ABA, *n* = 99; *klu* mock, *n* = 98; *klu* 5 μM ABA, *n* = 79; *klu* 10 μM ABA, *n* = 100; *35S:KLU-1* mock, *n* = 80; *35S:KLU-1* 5 μM ABA, *n* = 99; *35S:KLU-1* 10 μM ABA, *n* = 99. B, WT mock, *n* = 99; WT 5 μM ABA, *n* = 99; WT 10 μM ABA, *n* = 100; *klu* mock, *n* = 100; *klu* 5 μM ABA, *n* = 100; *klu* 10 μM ABA, *n* = 99; *35S:KLU-2* mock, *n* = 100; *35S:KLU-2* 5 μM ABA, *n* = 100; *35S:KLU-2* 10 μM ABA, *n* = 100). (C, D) Relative stomatal aperture compared to those treated without ABA. Different letters indicate statistically significant differences (P < 0.05) by one-way ANOVA followed by a Tukey's post-hoc test.
