# Supplementary figures and images for: Multi-omics approach reveals the contribution of KLU to leaf longevity and drought tolerance
Source: Plant Physiol. 2020 Nov 28;185(2):352–68. doi: 10.1093/plphys/kiaa034 (PMC8133585; doi:10.1093/plphys/kiaa034)

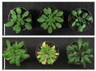

Supplement: kiaa034_Supplementary_Data [file kiaa034_supplementary_data.zip › pp.01345.2020-s04.jpg]
